# Predicting Outcomes From Radical Radiotherapy for Non-small Cell Lung Cancer: A Systematic Review of the Existing Literature

**DOI:** 10.3389/fonc.2018.00433

**Published:** 2018-10-10

**Authors:** Gerard M. Walls, Gerard G. Hanna, Fang Qi, Sai Zhao, Jun Xia, Mohammed T. Ansari, David Landau

**Affiliations:** ^1^Centre for Cancer Research & Cell Biology, Queen's University Belfast, Belfast, United Kingdom; ^2^Cancer Centre Belfast City Hospital, Belfast, United Kingdom; ^3^Systematic Review Solutions Ltd, The Ingenuity Centre, Nottingham, United Kingdom; ^4^Department of Global Health & Social Medicine, Faculty of Social Science & Public Policy, King's College London, London, United Kingdom; ^5^School of Epidemiology and Public Health, Faculty of Medicine, University of Ottawa, Ottawa, ON, Canada; ^6^Guy's and St Thomas' NHS Foundation Trust, London, United Kingdom; ^7^King's College London, Strand, London, United Kingdom

**Keywords:** radical radiotherapy, lung cancer, predictive biomarker, outcomes, literature map

## Abstract

Radical radiotherapy (RT) is a potentially curative treatment in non-small cell lung cancer (NSCLC) and is delivered in conventional 2-Gy fractions, hypofractionated and ablative stereotactic courses. No reliable, predictive biomarkers for the clinical events of local control, appearance of distant metastases and development of toxicity have been introduced in routine clinical practice. Such a test would enable the Radiotherapist to tailor the clinical management of individual patients, considering their pre-treatment characteristics, in order reduce the risk of recurrence or toxicity e.g., dose modification, accelerated fractionation, hypofractionation, or concurrent systemic therapy. The aim of this review was to map the published literature relating to investigations of the potential predictive value of patient or treatment characteristics in radical RT for NSCLC. These investigations should remain a research focus for disease control given the upward trends in lung cancer incidence, and for the avoidance of toxicity, given the survivorship afforded to the cohort of patients that do well with radical RT, or with the increasing range of systemic agents following metastatic relapse. The conclusion of the presented analysis is that there are no published, effective and validated predictive tools for estimation of risk of local/distant recurrence or toxicity after radical RT for NSCLC. The authors have identified an important space for future research in the field of lung cancer radiotherapy.

## Introduction

Radical radiotherapy (RT) is the term applied to describe a potentially curative dose of external beam RT for patients with non-small cell lung cancer (NSCLC) (1). The key clinical outcomes for patients undergoing radical RT are local tumor control, appearance of distant metastases and the development of treatment toxicity. Improvements in RT planning and delivery in recent years have resulted in its application to a broader range of patients, such as those with more advanced local disease (2) and those with early stage disease (3). Improvements in staging and systemic therapy are leading to longer overall survival in patients with locally advanced tumors (4). Despite these improvements and the emergence and adaptation of the technology permitting safe hypofractionated and ablative external beam radiotherapy, no reliable, predictive biomarkers for local control, appearance of distant metastases or development of toxicity have been introduced. To our knowledge, this important area of clinically unmet need has not been systematically reviewed. The purpose of this study, which may serve as a foundation from which to embark on dedicated research for radiotherapy researchers, is to summarize the currently available literature on predictors of the following three key clinical outcomes in treatment of NSCLC with RT:

1. Local control;
2. Metastases free survival;
3. Treatment related toxicity.

All studies including univariate or multivariate analyses for the effect of a patient or treatmeant characteristic on any of the 3 clinical outcomes of interest will be included in the analysis, or any study seeking to apply a proposed model. The authors would seek to establish a feasible line of investigation based on the existing literature if possible.

## Methods

### Literature mapping

Literature mapping was performed to retrieve, collate and map the variability found in the existing literature regarding predictors of response to radical RT treatment of inoperable NSCLC. Predictors of interest included were limited to:

- Prediction of which patients are likely to fail radical RT;
- Prediction of which patients are likely to benefit from particular types of (chemo)RT.

To be useful for clinical decision-making, predictive factors are defined as those covariates that are routinely available at baseline before the start of treatment, such as patient demographics, co-morbidities, disease characteristics, tumor markers, or radiological findings.

### Data sources

On July 17, 2017, EMBASE and MEDLINE were searched via Ovid SP and PubMed with no time/date, language, or document type limitations. The search strategy documented in Box 1 was developed using Medical Subject Headings (MeSH) for MEDLINE and Excerpta Medica Tree (EMTree) for EMBASE, by a team of clinicians and medical information specialists with database training.

Box 1 **Search strategies**

**EMBASE 1974 to 2017 Week 29**

1. Exp Non Small Cell Lung Cancer/ OR (((Bronch^*^ OR Lung OR Pulmon^*^) adj (“Non Small Cell?” OR “Nonsmall Cell?”) adj (Cancer^*^ OR Carcino^*^)) OR ((“Non Small Cell?” OR “Nonsmall Cell?”) adj (Bronchial OR Lung OR Pulmon^*^) adj (Cancer^*^ OR Carcino^*^)) OR NSCLC^*^).ti,ab.
2. Exp Radiotherapy/ OR (Radiotherap^*^ OR (Radiation adj Therap^*^)).ti,ab,fs.
3. Exp Prognosis/ OR Cohort Analysis/ OR Follow Up/ OR Incidence/ OR (Prognos^*^ OR Predict^*^ OR Outcome^*^ OR Cohort OR “Follow Up” OR Followup^*^ OR Longitudinal OR Prospective OR Incidence).ti,ab.
4. Exp Survival/ OR Survival Analysis/ OR Kaplan Meier Method/ OR Survivor/ OR Cancer Survivor/ OR Exp Toxicity Testing/ OR Cancer Grading/ OR Cancer Staging/ OR (Toxic^*^ OR Local Tumo?r Control OR Surviv^*^ OR “Kaplan Meier” OR Model^*^ OR ((Gleason OR Cancer OR Tumo?r OR TNM) adj (Stag^*^ OR Grad^*^ OR Scor^*^))).ti,ab. OR (Toxicity).fs.
5. 1 AND 2 AND 3 AND 4
6. Exp Animals/ OR Exp Invertebrate/ OR Animal Experiment/ OR Animal Model/ OR Animal Tissue/ OR Animal Cell/ OR Nonhuman/
7. Human/ OR Normal Human/ OR Human Cell/
8. 6 AND 7
9. 6 NOT 8
10. 5 NOT 9
11. Limit 10 to EMBASE

**MEDLINE**

1. “Carcinoma, Non-Small-Cell Lung”/ OR (((Bronch^*^ OR Lung OR Pulmon^*^) adj (“Non Small Cell?” OR “Nonsmall Cell?”) adj (Cancer^*^ OR Carcino^*^)) OR ((“Non Small Cell?” OR “Nonsmall Cell?”) adj (Bronchial OR Lung OR Pulmon^*^) adj (Cancer^*^ OR Carcino^*^)) OR NSCLC^*^).ti,ab.
2. Exp Radiotherapy/ OR (Radiotherap^*^ OR (Radiation adj Therap^*^)).ti,ab,fs.
3. Prognosis/ OR Exp Cohort Studies/ OR Follow-Up Studies/ OR Incidence/ OR (Prognos^*^ OR Predict^*^ OR Outcome^*^ OR Cohort OR “Follow Up” OR Followup^*^ OR Longitudinal OR Prospective OR Incidence).ti,ab.
4. Survival/ OR Survivors/ OR Exp Survival Analysis/ OR Survival Rate/ OR Disease-Free Survival/ OR Exp Toxicity Tests/ OR Neoplasm Grading/ OR Neoplasm Staging/ OR (Toxic^*^ OR Local Tumo?r Control OR Surviv^*^ OR “Kaplan Meier” OR Model^*^ OR ((Gleason OR Cancer OR Tumo?r OR TNM) adj (Stag^*^ OR Grad^*^ OR Scor^*^))).ti,ab. OR (Toxicity).fs.
5. 1 AND 2 AND 3 AND 4
6. 5 NOT (Animals NOT (Humans and Animals)).sh.
7. Limit 6 to MEDLINE

**PubMed**

(((Bronchial[tiab] OR Lung[tiab] OR Pulmon^*^[tiab]) AND (“Non Small Cell”[tiab] OR “Nonsmall Cell”[tiab]) AND (Cancer^*^[tiab] OR Carcino^*^[tiab])) OR NSCLC^*^[tiab]) AND (Radiotherap^*^[tiab] OR (Radiation[tiab] AND Therap^*^[tiab])) AND (Prognos^*^[tiab] OR Predict^*^[tiab] OR Outcome^*^[tiab] OR Cohort[tiab] OR “Follow Up”[tiab] OR Followup^*^[tiab] OR Longitudinal[tiab] OR Prospective[tiab] OR Incidence[tiab]) AND (Toxic^*^[tiab] OR “lOCAL Tumor Control”[tiab] OR “Local Tumour Control”[tiab] OR Surviv^*^[tiab] OR “Kaplan Meier”[tiab] OR Model^*^[tiab] OR ((Gleason[tiab] OR Cancer[tiab] OR Tumor[tiab] OR Tumour[tiab] OR TNM[tiab]) AND (Stag^*^[tiab] OR Grad^*^[tiab] OR Scor^*^[tiab]))) NOT MEDLINE[sb]

### Study selection

Two reviewers independently screened titles and abstracts of retrieved reports following deletion of obvious duplicates. Full texts of studies that passed title/abstract screening were then reviewed in detail to confirm eligibility. Randomized, non-randomized studies and systematic reviews investigating predictive factors or risk prediction models linked to response rate, loco-regional control, metastatic relapse, or toxicity were included where patients had stage I-III NSCLC and underwent radical RT with or without sequential, concurrent or adjuvant chemotherapy. All types of radical RT were included: conventional 2-Gy fractions, hypofractionated, and ablative stereotactic courses.

Manuscripts that were not available in full text, reported in a language other than English, small in sample size (< 50 patients), relating to outcomes not listed above or examining predictive factors not available before beginning radical RT were excluded. Studies including radiation and dosimetric parameters as predictive factors were not selected for analysis, except where they were part of a final risk prediction model. These and other treatment variables such as fractionation and RT technique would be better evaluated in a dedicated analysis of treatment factors as predictive biomarkers, and thus are not presented in this study.

One reviewer extracted data, and a sample (20%) of included studies were double-checked and verified for accuracy to confirm a satisfactory standard of extraction. Extraction included sample size, study design and general characteristics, population and tumor characteristics, radiotherapy and chemotherapy protocol, predicted outcomes and statistically significant risk factors. Adjusted estimates of association were extracted preferentially, with lack of adjustment included in assessment of risk of bias.

To assess the risk of bias, screening questions were posed about the suitability of the study design for testing of treatment and risk factor interaction, risk of selection bias, important confounding, and attrition bias. Studies were grouped into high, moderate and low risk of bias with supporting evidence on the data extraction form.

Risk factor association studies confined to a single treatment or where treatment response was not compared between intervention and control groups were not included as these do not estimate risk of treatment response (5). If a factor is associated with an outcome in both treated and untreated patients, the factor cannot be considered as a predictor of response to treatment and is best characterized as a prognosticator of the course of illness with respect to that particular outcome.

For risk prediction models we critically appraised the studies according to the American Joint Committee on Cancer Acceptance Criteria for Inclusion of Risk Models for Individualized Prognosis in the Practice of Precision Medicine (6). Models discriminating higher vs. lower risk patients were also assessed for validity to predict a differential relative or absolute benefit from treatment compared with an alternative option (5).

### Data coding

The following factors were recorded from the manuscripts reviewed:

1) Funding
Industry funding was recorded where full or partial for-profit funding was declared by the authors.
2) Study design
Included studies were categorized as follows:

#### Predictive factor research (univariate analysis)

Patient, tumor, treatment, or biomarker factor and the association with a given outcome.

#### Predictive model research (multivariate analysis)

Studies may be model derivation studies, model validation (internal or external) studies, or both. Those given alternative titles such as prediction index or rule were also included.

#### Predictive impact studies

Studies evaluate the effect of using a predictive model on patient outcomes. Such studies require a parallel control group of standard care or involve a time-series type design. Predictive impact studies evaluate the impact of a model on decision-making and patient outcomes. Methodological evaluation of these impact studies is similar to evaluation of effectiveness of drugs/devices/procedures.

Study design categorization was based both on the conduct of the study and the analytic approach for risk prediction. For example, a comparative study may have analyze the outcome data pooled from all interventional groups using a multi/univariate regression analysis with treatment as a covariate, but because estimate of differential treatment effect was not the dependent factor for risk prediction, the study was characterized as non-comparative.

3) Predictive factors
Considerable heterogeneity was discovered in reported risk factors across studies. The following variables were extracted:
Chemotherapy: concurrent vs. sequential or no chemotherapy; specific regimens vs. other regimens
Comorbidity: classification scores, interstitial lung disease, COPD, vascular embolism, necrotic tumors
Contouring: gross tumor volume
Fractionation: hyperfractionation, hypofractionation, conventional fractionation, inter-fraction interval
Imaging: coarseness, contrast, busyness, apparent diffusion coefficient
Lung function: transfer factor, total lung capacity, diffusing capacity
Medications: β-blockers, ACE inhibitors
Molecular markers: single nucleotide polymorphisms and tumor markers
Performance status: ECOG or KPS
PET data: maximum standard uptake value; retention index, metabolic tumor volume, tumor growth rate, total lesion glycolysis
RT technique: 2D conventional, 3D-CT, IMRT, Arc therapy, stereotactic
Stage: clinical, tumor dimensions, PET-CT findings, final TNM stage
Trace elements (hair): Au and Cu

4) Fractionation
Conventional RT
Total dose 50–55 Gy, in approx. 20 fractions or approx. 2 Gy/fraction
Conventional RT, dose intensified
Total dose >55 Gy when given in 20 fractions or total dose >66 Gy when reported as 2G per fraction
Hypofractionation
Investigator defined. Employs fewer total fractions but increased dose per fraction.
Hyperfractionation
Investigator defined. RT is given more than once per day. Total RT dose may be lower or higher than conventional schedules.

## Results

The results section has been divided into characteristics of studies and the patients included, summarized publications relating to toxicity, summarized publications relating to local/distant disease control, and summarized publications relating to predictive model validation.

### Study characteristics

**Figure 1:**
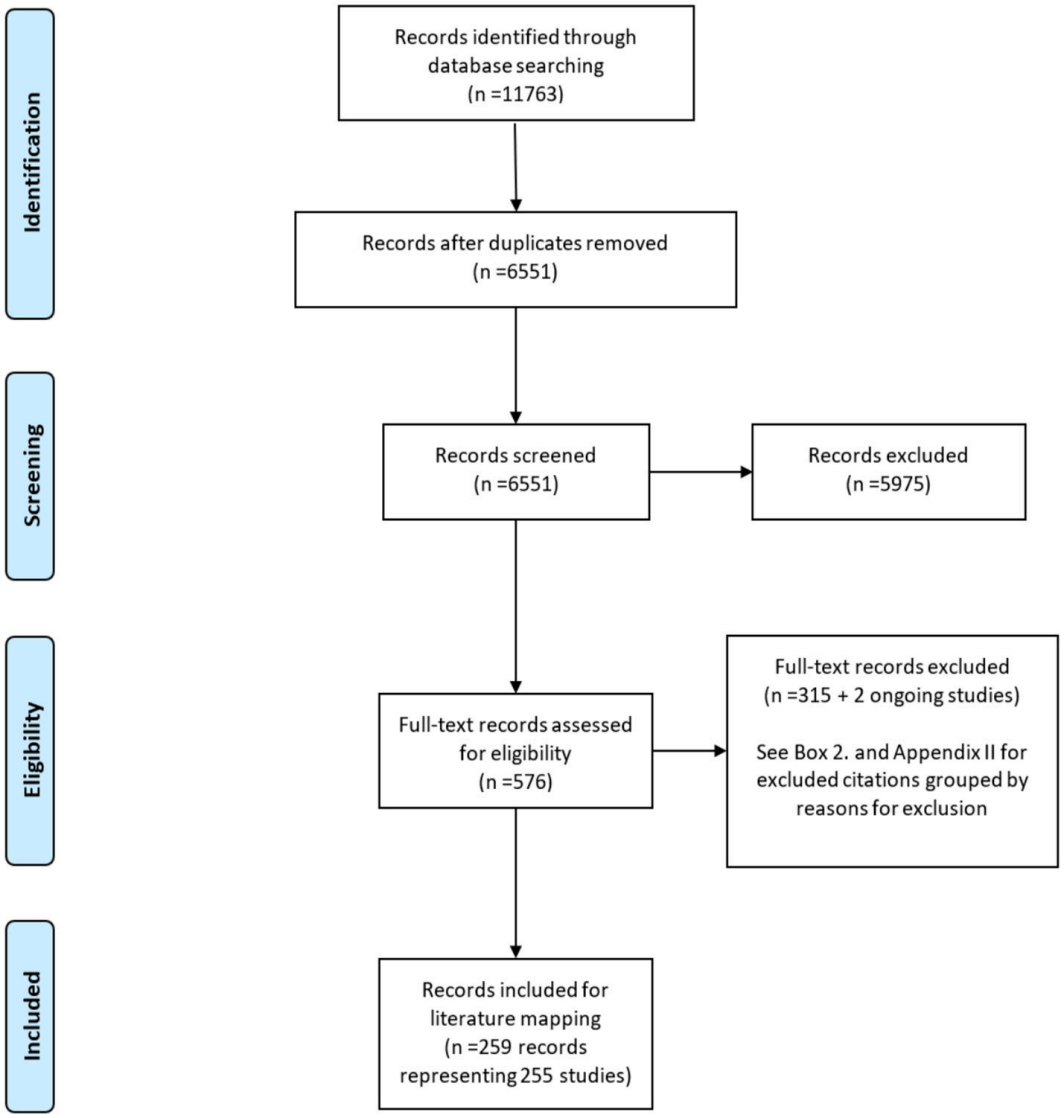
PRISMA flow diagram.

The PRISMA flow diagram in Figure 1 illustrates the flow of 7320 reports retrieved in literature searches as they were screened for eligibility. A total of 259 reports were finally included (see Appendix A in Supplementary Material). Excluded studies were categorized by exclusion criterion listed in Box 2 (see Appendix B in Supplementary Material). Approximately 63% of included reports were published in the period 2010–2016 (see Appendix C in Supplementary Material, Figure 1).

Box 2 **Reasons for exclusion of reports.**

(1) Sample size < 50, N = 8
(2) Population was not predominantly (>95% of population) stage IA, IB, IIA, IIB, IIIA, and/or IIIB NSCLC or an analyzed subgroup of these stages, *N* = 72
(3) Treatment was not radical RT – patients had surgery before radiotherapy, *N* = 36
(4) Did not investigate risk factor(s) and radical RT association, *N* = 5
(5) Did not clinical impact (effectiveness and harms) of response to radical RT risk prediction guided patient management, *N* = 1
(6) Language other than English, *N* = 45
(7) Record is a non-systematic review, letter, editorial, or case-report, *N* = 19
(8) Investigation is about average risk/incidence/odds of specific outcomes following radical RT (i.e., fundamental prognosis research) but not about predictive factor risk assessment, *N* = 1
(9) Predicted outcome is not a pre-specified outcome, *N* = 77
(10) Reports excluded because none of the risk factors evaluated are factors that were measured at or before the start of radical RT, *N* = 3
(11) Reports excluded because evaluated radical RT dose (but not the modality of radical RT) as the only risk factor for response to treatment, *N* = 16
(12) Model development methods paper using radiotherapy for NSCLC as an example; results not intended as formal clinical findings, *N* = 2
(13) Unclear record, *N* = 30

The majority of reports described reports of prognostic factors as opposed to investigations of predictors of outcome. Most studies were of non-comparative, single-arm design, precluding assessment of prediction of differential response to radical RT. The few comparative studies that compared one technique, fractionation, dose, or chemoradiotherapy protocol with another, did not analyse the data in a manner that would permit investigation of predictors of relative treatment effect as conceptualized in the Introduction.

Ten risk prediction models were also identified in the included body of evidence. Several of these were also categorized as prognostic factor research. Only one prognostic model was externally validated and further refined and no model was validated for predicting response to therapy.

Hingorani et al. suggest that validation of models predictive of response to treatment includes estimation of individual risk of patients, categorizing patients into higher and lower risk groups, and establishing that relative or absolute benefits from a treatment are distinctly different for higher and lower risk patients (5). As such, all model development studies and the vast majority of predictive factor research identified were considered to be of poor quality with respect to the objective of predicting response to radical RT.

The 255 included studies (comprising 259 reports) were based on data from 71,993 patients. However, the true number of included patients is likely to be slightly lower due to the inclusion of some companion reports that were not identified as such. Single-center studies comprised 66% of the included manuscripts, multi-center comprised 22%, and the remainder did not report. Industry sponsorship was documented for 8% included studies, non-profit organization sponsorship for 39% and 53% studies did not include these details. Most studies (95%) were conducted in North America, Europe or Asia.

Randomized controlled trials comprised only 8% included studies and a further 3% included studies were comparative but not randomized, but the majority (90%) of identified studies were non-comparative. Two systematic reviews were identified as meeting the inclusion criteria. Risk of bias was deemed high in 65% randomized controlled trials, 100% of non-randomized comparative studies and 100% non-comparative studies.

A majority of studies included patients with stage III disease (exclusively, or with stage I/II) of a range of ages and tumor histology (Table 1). Performance status was not reported in 19% of reports, while over 60% of studies included patients a performance status of ECOG ≤ 2 or KPS ≥ 60.

**Table 1:** Mapping of study population and tumor characteristics (*n* = 259).

**Item**	**Variables**	***N***	**%**	**Item**	**Variables**	***N***	**%**
Tumor stage				Tumor histology			
	I only	40	15		Squamous cell CA	2	1
	III only	100	39		Non-squamous cell CA	7	3
	Stage I-III (mixed)	64	25		Mixed squamous and non-squamous cell CA	209	81
	Other combinations (stage I &III, I & II, and II & III)	55	21		NR	41	16
Staging	
	PET-staged	49	19	Age			
	PET or CT-staged	20	8		Predominantly or exclusively elderly (>65 y)	12	5
	Non-PET staged	59	23		Non-elderly only (≤ 65 y)	0	0
	NR	131	51		Mixed elderly and non-elderly ages	233	90
Performancestatus					NR	14	5
	Predominantly (≥80% of sample) ≤ 2 (ECOG) OR ≥ 60 (KPS)	164	63				
	Unclear or other distribution	46	18				
	NR	49	19				

*CA, carcinoma; N, number of study reports; NR, not reported; PET, positron emission tomography; CT, computerized tomography; NR, not reported; ECOG, Eastern Cooperative Oncology Group; KPS, Karnofsky performance status*.

Radical RT techniques commonly described in the studies included 3D-conformal, IMRT, and stereotactic ablative radiotherapy (see Appendix C in Supplementary Material, Table 1). Fractionation varied widely between the included studies with no clearly preferred schedule. Radical RT was reported to have been administered without chemotherapy in 31% of included reports, concurrently with chemotherapy in 13% and sequentially in 3% (see Appendix C in Supplementary Material, Table 2).

### Predictors of toxicity with radical RT

Radical RT toxicity outcomes were reported in 43% of studies (Table 2). Of the reports demonstrating a positive association between a factor and acute oesophageal or lung toxicity outcomes, no marker was found to have a consistent prognostic value, reflecting variability in the design of studies, findings, or both. Across studies contributing data for any one of the toxicity outcomes of interest, chemotherapy was the most commonly reported statistically significant risk factor (20% of 112 reports). Age, gender, performance status, tumor stage, fractionation, radiotherapy technique and molecular markers were found to be significant risk factors ≤ 15% of reports investigating a toxicity outcome.

**Table 2:** Mapping of statistically significant predictors of radiotherapy toxicity.

**Outcome (*N* reports)**	**Patient factors**	**%**	**Tumor factors**	**%**	**Treatment factors**	**%**	**% of reports with no significant predictor**
Oesophagitis (37)							65
	Age	5	Stage	3	Chemotherapy	24	
	Gender	3			Fractionation	3	
	Performance status	3			RT technique	8	
	Race	5					
	Molecular marker (SNP)	5					
Oesophageal stricture (1)							100
Composite oesophageal toxicity (18)							61
	Gender	6	Stage	11	Chemotherapy	28	
	Symptoms	6					
	Molecular marker (TGF-β)	6					
	Weight loss	17					
Radiation pneumonitis (58)							64
	Age	9	PET data	2	Chemotherapy	10	
	Comorbidity	3	PTV	5	RT technique	3	
	Gender	2	Stage	5	RT fractionation	2	
	Lung function	7	Tumor site	3			
	Lung volume	2					
	Medications	2					
	Performance status	7					
	Smoking	9					
	Molecular markers (APEX1, AT1, Protein, SNP, TGF-β, TNF, VEGF, XRCC1, XRCC3)	10					
Lung fibrosis (2)							100
Composite lung toxicity (27)							70
	Age	15	Histology (large cell vs. adenocarcinoma)	4	Chemotherapy	7	
	Comorbidity	11			Fractionation (N of fractions)	4	
	Gender	4	Stage	11	Treatment center	4	
	Lung function	4	Tumor site	7	RT Technique	7	
	Performance status	4					
	Race	4					
	Weight loss	7					
Composite acute toxicities (1)							0
			Tumor site	100			
Death due to RT toxicity (4)							50
			Tumor site	50			

*Identical study reports may be counted for more than one predictor therefore total percentage may not be equal to 100*.

*AT, angiotensin receptor; N, number of; PTV, planning target volume; RT, radiotherapy; SNP, single-nucleotide polymorphism; TGF-β, transforming growth factor beta; TNF, tumor necrosis factor; VEGF, vascular endothelial growth factor; vs, versus*.

### Predictors of local/distant control with radical RT

One hundred and eighty-one reports investigated the association between a risk factor and local tumor control, defined inconsistently in the included reports (Table 3). Almost 50% of these studies found no statistically significant association. Of the reported risk factors that were significantly associated with local control, most common were tumor stage, performance status and chemotherapy administration.

**Table 3:** Mapping of statistically significant predictors of local or distant tumor control.

**Outcome (N)**	**Patient factors**	**%**	**Tumor factors**	**%**	**Treatment factors**	**%**	**% of reports with no significant predictor**
**LOCAL CONTROL (outcomes: disease free survival, disease progression, failure free survival, local failure, local progression free survival, local or disease recurrence, nodal failure, non-local relapse free survival, primary tumor control, time to progression, tumor growth, or tumor shrinkage)**							
181							49
	Age	6	Date (staging after PET scan introduction)	< 1	Booster field size	< 1	
	Blood marker (neutrophil/lymphocyte ratio)	< 1	Histology (SCC or lymphovascular invasion)	7	Chemotherapy	12	
	Gender	9	Imaging (texture)	2	Contouring	2	
	Lung function	< 1	PET data	8	Field size	< 1	
	Medication	< 1	PTV	< 1	Fractionation	3	
	Molecular markers (e.g., AI, Bcl-2, COX2, EGFR, FasL, FGF-2, HER-2, MMP-2, p53, SPARC expression, Rb, trace elements, VEGF, etc.)	7	Stage	34	RT technique	5	
	Performance status	17	Tissue(clinical vs. pathological diagnosis)	< 1			
	Smoking	2	Total tumor volume	2			
	Symptoms	1	Tumor site	3			
	Weight loss	4					
**DISTANT CONTROL (outcomes: metastatic disease-free survival, distant metastasis, or distant failure)**							
72							46
	Age	6	Histology (SCC, lymphovascular invasion, or tumor grade)	11	Chemotherapy	3	
	Blood marker (platelet-to-lymphocyte ratio)	1	PET data	8	Contouring	1	
	Comorbidity	4	Stage	24	Fractionation	4	
	Gender	8	Total tumor volume	1	RT Technique	1	
	Medication	1	Tumor site	4			
	Molecular markers (e.g., SNP, apoptotic index, index based on CRP, albumin, etc.)	7					
	Performance status	15					
	Weight loss	6					
**BRAIN METASTASIS**							
8							50
	Age	25	Histology (SCC or tumor grade)	38	Chemo	13	
	Molecular marker (neuron-specific enolase or CA125)	25	Tumor marker	13			
	Performance status	13					

*Identical study reports may be counted for more than one predictor therefore total percentage may not be equal to 100*.

*AI, Apoptotic index; EGFR, epidermal growth factor receptor; FGF-2, fibroblast growth factor-2; HER-2, human epidermal growth factor receptor-2; MMP-2, matrix metalloproteinase; Rb, retinoblastoma protein; VEGF, vascular endothelial growth factor; SCC, squamous cell carcinoma; PTV, planning target volume; RT, radiotherapy*.

Distant tumor control was investigated in 72 reports, with almost half reporting no significant association with any risk factor (Table 3). Of the reported risk factors that were significantly associated with distant control, most common were tumor stage, performance status, and tumor histology.

Studies were generally not designed to investigate predictors of response to radical RT. All risk factors for which a statistically significant association was found were prognostic factors predicting the risk of outcomes following (chemo)RT. Whether these factors could also guide treatment selection remains to be established.

### Prognostic models predicting treatment response

Included reports were model development or validation studies in 10 of 259 cases. Sample size ranged from 54 to 836 patients. No model was validated for prediction of response to treatment and therefore all models were considered inappropriate for guiding patient selection for specific treatments. A vast range of predicted outcomes were noted in these studies (see Appendix D in Supplementary Material).

## Discussion

Prognostic research predicts the course of illness, not response to therapy. Prediction of response to treatment guides appropriate treatment selection based on risk characteristics shared by subgroups of patients. When the relative treatment effect is the approximately the same for all subgroups of patients, treatment decisions are best guided by patients' individual baseline risk for outcomes. Baseline risk is evaluated in prognostic risk factor and prognostic model research, which are distinct from predictive factor research investigating differential treatment response. Patient groups with a higher baseline risk will have greater absolute benefits than patients with a lower baseline risk.

This mapping exercise highlights a clear paucity of predictive factor research, an important component of stratified medicine, in the setting of radical RT for NSCLC (5). Parameters that have been considered as predictive biomarkers in the literature analyzed include patient demographics, initial staging and radiomic and dosimetric correlates. This limited available published evidence was found to be at high risk of confounding from various biases (see Appendix D in Supplementary Material) however, meaning these signals must be interpreted with caution. It may be that they provide a framework with which to begin new workstreams of radical RT research. The evidence base in NSCLC patients undergoing radical RT is of the prognostic type predominantly. The presented results are in keeping with the previous observation that most risk estimation is of a prognostic type, rather than predictive of response to therapy (5, 7).

The presented review exposes the gross uncoupling of radiation and medical oncology in the implementation of biomarker-informed clinical decision making. An array of technical overtures have reformed radiation oncology in recent decades, leading to gains in tumor control and treatment toxicity predominantly through improved accuracy (8). However systemic therapy for lung cancer has been revolutionized for the subsets of patients with an EGFR mutation, ALK rearrangement or PD-L1 positivity by targeted agents which are initiated in appropriate clinical scenarios and based on widely available predictive biomarkers (9). There is increasing appreciation amongst academic radiation oncologists and clinician scientists that such novel biology-based discovery and translation can only result from an intense research focus in radiotherapy. New collaborative groups with this ethos are currently striving to address the illustrated dearth of predictive biomarkers in radiotherapy (10).

It is important for patients with NSCLC that more personalized radiotherapy approaches are developed rapidly, given the disappointing outcomes experienced on the whole in this difficult disease. Future research on truly predictive factors needs to focus in two directions. Firstly, more precise markers of individual response and risk need to be developed. These might include molecular biomarkers for radiosensitivity in both tumor and patient, immune host markers and fuller imaging analysis such as radiomics. In parallel, trial design must be adapted so that individual factors can be assessed for their relationship with a differential trial arm outcome for assessment of predictive value.

## Author contributions

GW: study design, collation of results, and drafting manuscript; GH and MA: study design, data collection, collation of results, and drafting manuscript; FQ, SZ, and JX: data collection, collation of results, and drafting manuscript; DL: conception, study design, data collection, collation of results, and drafting manuscript.

### Conflict of interest statement

FQ, SZ, and JX are employed by Systematic Review Solutions Ltd. MA is contracted as a Consultant by Systematic Review Solutions Ltd. The remaining authors declare that the research was conducted in the absence of any commercial or financial relationships that could be construed as a potential conflict of interest.

## References

[1] Jiang ZQ Yang K Komaki R Wei X Tucker SL Zhuang Y. Long-term clinical outcome of intensity-modulated radiotherapy for inoperable non-small cell lung cancer: the MD Anderson experience. Int J Radiat Oncol Biol Phys. (2012) 83:332–9. 10.1016/j.ijrobp.2011.06.1963 22079735

[2] Christodoulou M Bayman N McCloskey P Rowbottom C Faivre-Finn C. New radiotherapy approaches in locally advanced non-small cell lung cancer. Eur J Cancer (2014) 50:525–34. 10.1016/j.ejca.2013.11.027 24333095

[3] O'Rourke N Roqué I Figuls M Farré Bernadó N Macbeth F Concurrent chemoradiotherapy in non-small cell lung cancer. Cochrane Database Syst Rev. (2010) CD002140 10.1002/14651858.CD002140.pub3 20556756 PMC12584098

[4] Liao ZX Komaki RR Thames HD Liu HH Tucker SL Mohan R. Influence of technologic advances on outcomes in patients with unresectable, locally advanced non-small-cell lung cancer receiving concomitant chemoradiotherapy. Int J Radiat Oncol Biol Phys. (2010) 76:775–81. 10.1016/j.ijrobp.2009.02.032 19515503

[5] Hingorani AD van der Windt AD Riley RD Abrams K Moons KGM Steyerberg EW et al. Prognosis research strategy (PROGRESS) 4: stratified medicine research. BMJ (2013) 346:e5793. 10.1136/bmj.e5793 23386361 PMC3565686

[6] Kattan MW Hess KR Amin MB Lu Y Moons KG Gershenwald JE. American Joint Committee on Cancer acceptance criteria for inclusion of risk models for individualized prognosis in the practice of precision medicine. Cancer J Clin. (2016) 66:370–4. 10.3322/caac.21339 26784705 PMC4955656

[7] Clark GM. Prognostic factors versus predictive factors: examples from a clinical trial of erlotinib. Mol Oncol. (2008) 1:406–12. 10.1016/j.molonc.2007.12.001 19383314 PMC5543832

[8] Choudhury A Budgell G MacKay R Falk S Faivre-Finn C Dubec M. The future of image-guided radiotherapy. Clin Oncol. (2017) 29:662–6. 10.1016/j.clon.2017.04.036 28511968

[9] Sun Y Xu J Zhou J Liu WJ. Targeted drugs for systemic therapy of lung cancer with brain metastases. Oncotarget (2018) 9:5459–72. 10.18632/oncotarget.23616 29435193 PMC5797064

[10] Hanna G McDonald F Greystoke A Forester M Brown S Hall E EP-1228: UK NCRI CTRad consensus on drug and radiotherapy combination platform studies in NSCLC. Radiother Oncol. (2017) 123(Suppl 1):S662–3. 10.1016/S0167-8140(17)31663-8

