# Corrigendum: Predicting Outcomes From Radical Radiotherapy for Non-small Cell Lung Cancer: A Systematic Review of the Existing Literature

**DOI:** 10.3389/fonc.2019.00112

**Published:** 2019-03-01

**Authors:** Gerard M. Walls, Gerard G. Hanna, Fang Qi, Sai Zhao, Jun Xia, Mohammed T. Ansari, David Landau

**Affiliations:** ^1^Centre for Cancer Research & Cell Biology, Queen's University Belfast, Belfast, United Kingdom; ^2^Cancer Centre Belfast City Hospital, Belfast, United Kingdom; ^3^Systematic Review Solutions Ltd, The Ingenuity Centre, Nottingham, United Kingdom; ^4^Department of Global Health & Social Medicine, Faculty of Social Science & Public Policy, King's College London, London, United Kingdom; ^5^School of Epidemiology and Public Health, Faculty of Medicine, University of Ottawa, Ottawa, ON, Canada; ^6^Guy's and St Thomas' NHS Foundation Trust, London, United Kingdom; ^7^King's College London, Strand, London, United Kingdom

**Keywords:** radical radiotherapy, lung cancer, predictive biomarker, outcomes, literature map

In the original article, there was an error. The Conflict of Interest statement used was incorrect.

A correction has been made to the Conflict of Interest Statement:

“FQ, SZ, and JX are employed by Systematic Review Solutions Ltd. MA is contracted as a Consultant by Systematic Review Solutions Ltd.

The remaining authors declare that the research was conducted in the absence of any commercial or financial relationships that could be construed as a potential conflict of interest.”

The authors apologize for this error and state that this does not change the scientific conclusions of the article in any way. The original article has been updated.

