# Supplementary material for: Predicting Outcomes From Radical Radiotherapy for Non-small Cell Lung Cancer: A Systematic Review of the Existing Literature
Source: Front Oncol. 2018 Oct 10;8:433. doi: 10.3389/fonc.2018.00433 (PMC6191477; doi:10.3389/fonc.2018.00433)
Supplement: Supplementary file 1 [file Data_Sheet_1.DOCX]

Appendix A

References of included studies

1. Saynak, M., et al., *The results of concomitant and sequential chemoradiotherapy with cisplatin and etoposide in patients with locally advanced non-small cell lung cancer.* Journal of B.U.On., 2005. 10(2): p. 213-8.
2. Kong, F.M., et al., *Final toxicity results of a radiation-dose escalation study in patients with non-small-cell lung cancer (NSCLC): predictors for radiation pneumonitis and fibrosis.* International Journal of Radiation Oncology, Biology, Physics, 2006. 65(4): p. 1075-86.
3. Hoppe, B.S., et al., *Acute skin toxicity following stereotactic body radiation therapy for stage I non-small-cell lung cancer: who's at risk?* International Journal of Radiation Oncology, Biology, Physics, 2008. 72(5): p. 1283-6.
4. Moreno-Jimenez, M., et al., *Dosimetric analysis of the patterns of local failure observed in patients with locally advanced non-small cell lung cancer treated with neoadjuvant chemotherapy and concurrent conformal (3D-CRT) chemoradiation.* Radiotherapy & Oncology, 2008. 88(3): p. 342-50.
5. Henderson, M., et al., *Baseline pulmonary function as a predictor for survival and decline in pulmonary function over time in patients undergoing stereotactic body radiotherapy for the treatment of stage I non-small-cell lung cancer.* International Journal of Radiation Oncology, Biology, Physics, 2008. 72(2): p. 404-9.
6. Tsakiridis, T., et al., *Association of phosphorylated epidermal growth factor receptor with survival in patients with locally advanced non-small cell lung cancer treated with radiotherapy.* Journal of Thoracic Oncology: Official Publication of the International Association for the Study of Lung Cancer, 2008. 3(7): p. 716-22.
7. Kuyama, S., et al., *Impact of HER2 gene and protein status on the treatment outcome of cisplatin-based chemoradiotherapy for locally advanced non-small cell lung cancer.* Journal of Thoracic Oncology: Official Publication of the International Association for the Study of Lung Cancer, 2008. 3(5): p. 477-82.
8. Nakamura, T., et al., *Clinical outcome of stage III non-small-cell lung cancer patients after definitive radiotherapy.* Lung, 2008. 186(2): p. 91-6.
9. Werner-Wasik, M., et al., *Increasing tumor volume is predictive of poor overall and progression-free survival: secondary analysis of the Radiation Therapy Oncology Group 93-11 phase I-II radiation dose-escalation study in patients with inoperable non-small-cell lung cancer.* International Journal of Radiation Oncology, Biology, Physics, 2008. 70(2): p. 385-90.
10. Semrau, S., et al., *Impact of comorbidity and age on the outcome of patients with inoperable NSCLC treated with concurrent chemoradiotherapy.* Respiratory Medicine, 2008. 102(2): p. 210-8.
11. Moreno, M., et al., *Predictive factors for radiation-induced pulmonary toxicity after three-dimensional conformal chemoradiation in locally advanced non-small-cell lung cancer.* Clinical & Translational Oncology: Official Publication of the Federation of Spanish Oncology Societes & of the National Cancer Institute of Mexico, 2007. 9(9): p. 596-602.
12. Hartsell, W.F., et al., *Can serum markers be used to predict acute and late toxicity in patients with lung cancer? Analysis of RTOG 91-03.* American Journal of Clinical Oncology, 2007. 30(4): p. 368-76.
13. Zhao, L., et al., *High radiation dose may reduce the negative effect of large gross tumor volume in patients with medically inoperable early-stage non-small cell lung cancer.* International Journal of Radiation Oncology, Biology, Physics, 2007. 68(1): p. 103-10.
14. Chen, T.F., et al., *CK19 mRNA expression measured by reverse-transcription polymerase chain reaction (RT-PCR) in the peripheral blood of patients with non-small cell lung cancer treated by chemo-radiation: an independent prognostic factor.* Lung Cancer, 2007. 56(1): p. 105-14.
15. Ishikawa, H., et al., *Effect of histologic type on recurrence pattern in radiation therapy for medically inoperable patients with stage I non-small-cell lung cancer.* Lung, 2006. 184(6): p. 347-53.
16. Jeremic, B., et al., *Pretreatment prognostic factors in patients with early-stage (I/II) non-small-cell lung cancer treated with hyperfractionated radiation therapy alone.* International Journal of Radiation Oncology, Biology, Physics, 2006. 65(4): p. 1112-9.
17. Fokkema, E., et al., *Expression and prognostic implications of apoptosis-related proteins in locally unresectable non-small cell lung cancers.* Lung Cancer, 2006. 52(2): p. 241-7.
18. Kawaguchi, T., et al., *Second primary cancers in patients with stage III non-small cell lung cancer successfully treated with chemo-radiotherapy.* Japanese Journal of Clinical Oncology, 2006. 36(1): p. 7-11.
19. Chapet, O., et al., *Normal tissue complication probability modeling for acute esophagitis in patients treated with conformal radiation therapy for non-small cell lung cancer.* Radiotherapy & Oncology, 2005. 77(2): p. 176-81.
20. Belderbos, J., et al., *Acute esophageal toxicity in non-small cell lung cancer patients after high dose conformal radiotherapy.* Radiotherapy & Oncology, 2005. 75(2): p. 157-64.
21. Borst, G.R., et al., *Standardised FDG uptake: a prognostic factor for inoperable non-small cell lung cancer.* European Journal of Cancer, 2005. 41(11): p. 1533-41.
22. Qiao, W.B., et al., *Clinical and dosimetric factors of radiation-induced esophageal injury: radiation-induced esophageal toxicity.* World Journal of Gastroenterology, 2005. 11(17): p. 2626-9.
23. Chang, J.Y., et al., *High mutagen sensitivity in peripheral blood lymphocytes predicts poor overall and disease-specific survival in patients with stage III non-small cell lung cancer treated with radiotherapy and chemotherapy.* Clinical Cancer Research, 2005. 11(8): p. 2894-8.
24. Bradley, J., et al., *Toxicity and outcome results of RTOG 9311: a phase I-II dose-escalation study using three-dimensional conformal radiotherapy in patients with inoperable non-small-cell lung carcinoma.* International Journal of Radiation Oncology, Biology, Physics, 2005. 61(2): p. 318-28.
25. Jeremic, B., et al., *Interfraction interval in patients with stage III non-small-cell lung cancer treated with hyperfractionated radiation therapy with or without concurrent chemotherapy: final results in 536 patients.* American Journal of Clinical Oncology, 2004. 27(6): p. 616-25.
26. Jeremic, B., et al., *Stage III non-small-cell lung cancer treated with high-dose hyperfractionated radiation therapy and concurrent low-dose daily chemotherapy with or without weekend chemotherapy: retrospective analysis of 301 patients.* American Journal of Clinical Oncology, 2004. 27(4): p. 350-60.
27. Fox, J.L., K.E. Rosenzweig, and J.S. Ostroff, *The effect of smoking status on survival following radiation therapy for non-small cell lung cancer.* Lung Cancer, 2004. 44(3): p. 287-93.
28. Sibtain, A., et al., *Pre-treatment haemoglobin concentration in accelerated and conventional radiotherapy for non-small cell lung carcinoma.* Clinical Oncology (Royal College of Radiologists), 2004. 16(1): p. 58-62.
29. Schild, S.E., et al., *The outcome of combined-modality therapy for stage III non-small-cell lung cancer in the elderly.* Journal of Clinical Oncology, 2003. 21(17): p. 3201-6.
30. Langendijk, H., et al., *The importance of pre-treatment haemoglobin level in inoperable non-small cell lung carcinoma treated with radical radiotherapy.* Radiotherapy & Oncology, 2003. 67(3): p. 321-5.
31. Brooks, K.R., et al., *Measurement of chemoresistance markers in patients with stage III non-small cell lung cancer: a novel approach for patient selection.* Annals of Thoracic Surgery, 2003. 76(1): p. 187-93; discussion 193.
32. Bradley, J.D., et al., *Elective nodal failures are uncommon in medically inoperable patients with Stage I non-small-cell lung carcinoma treated with limited radiotherapy fields.* International Journal of Radiation Oncology, Biology, Physics, 2003. 56(2): p. 342-7.
33. Singh, A.K., M.A. Lockett, and J.D. Bradley, *Predictors of radiation-induced esophageal toxicity in patients with non-small-cell lung cancer treated with three-dimensional conformal radiotherapy.* International Journal of Radiation Oncology, Biology, Physics, 2003. 55(2): p. 337-41.
34. Etiz, D., et al., *Influence of tumor volume on survival in patients irradiated for non-small-cell lung cancer.* International Journal of Radiation Oncology, Biology, Physics, 2002. 53(4): p. 835-46.
35. Chen, M., et al., *Prognostic factors for local control in non-small-cell lung cancer treated with definitive radiation therapy.* American Journal of Clinical Oncology, 2002. 25(1): p. 76-80.
36. Bradley, J.D., et al., *Gross tumor volume, critical prognostic factor in patients treated with three-dimensional conformal radiation therapy for non-small-cell lung carcinoma.* International Journal of Radiation Oncology, Biology, Physics, 2002. 52(1): p. 49-57.
37. Madej, P.J., et al., *Combined modality therapy for Stage IIIMO non-small cell lung cancer. A five-year experience.* Cancer, 1984. 54(1): p. 5-12.
38. Schaake-Koning, C., et al., *Prognostic factors of inoperable localized lung cancer treated by high dose radiotherapy.* International Journal of Radiation Oncology, Biology, Physics, 1983. 9(7): p. 1023-8.
39. Shibamoto, Y., et al., *Influence of interfraction interval on the efficacy and toxicity of hyperfractionated radiotherapy in combination with concurrent daily chemotherapy in stage III non-small-cell lung cancer.* International Journal of Radiation Oncology, Biology, Physics, 2001. 50(2): p. 295-300.
40. Mac Manus, M.P., et al., *F-18 fluorodeoxyglucose positron emission tomography staging in radical radiotherapy candidates with nonsmall cell lung carcinoma: powerful correlation with survival and high impact on treatment.* Cancer, 2001. 92(4): p. 886-95.
41. Hwang, J.H., et al., *Apoptosis and bcl-2 expression as predictors of survival in radiation-treated non-small-cell lung cancer.* International Journal of Radiation Oncology, Biology, Physics, 2001. 50(1): p. 13-8.
42. Ataman, O.U., et al., *Failure-specific prognostic factors after continuous hyperfractionated accelerated radiotherapy (CHART) or conventional radiotherapy in locally advanced non-small-cell lung cancer: a competing risks analysis.* British Journal of Cancer, 2001. 85(8): p. 1113-8.
43. Langendijk, H., et al., *Cell proliferation and apoptosis in stage III inoperable non-small cell lung carcinoma treated by radiotherapy.* Radiotherapy & Oncology, 2000. 56(2): p. 197-207.
44. Chen, M., et al., *The impact of overall treatment time on outcomes in radiation therapy for non-small cell lung cancer.* Lung Cancer, 2000. 28(1): p. 11-9.
45. Werner-Wasik, M., et al., *Interfraction interval does not affect survival of patients with non-small cell lung cancer treated with chemotherapy and/or hyperfractionated radiotherapy: a multivariate analysis of 1076 RTOG patients.* International Journal of Radiation Oncology, Biology, Physics, 1999. 44(2): p. 327-31.
46. Nguyen, L.N., et al., *Effectiveness of accelerated radiotherapy for patients with inoperable non-small cell lung cancer (NSCLC) and borderline prognostic factors without distant metastasis: a retrospective review.* International Journal of Radiation Oncology, Biology, Physics, 1999. 44(5): p. 1053-6.
47. Movsas, B., et al., *The benefit of treatment intensification is age and histology-dependent in patients with locally advanced non-small cell lung cancer (NSCLC): a quality-adjusted survival analysis of radiation therapy oncology group (RTOG) chemoradiation studies.* International Journal of Radiation Oncology, Biology, Physics, 1999. 45(5): p. 1143-9.
48. Martel, M.K., et al., *Estimation of tumor control probability model parameters from 3-D dose distributions of non-small cell lung cancer patients.* Lung Cancer, 1999. 24(1): p. 31-7.
49. Maguire, P.D., et al., *Clinical and dosimetric predictors of radiation-induced esophageal toxicity.* International Journal of Radiation Oncology, Biology, Physics, 1999. 45(1): p. 97-103.
50. Machtay, M., et al., *Is prolonged survival possible for patients with supraclavicular node metastases in non-small cell lung cancer treated with chemoradiotherapy?: Analysis of the Radiation Therapy Oncology Group experience.* International Journal of Radiation Oncology, Biology, Physics, 1999. 44(4): p. 847-53.
51. Sibley, G.S., et al., *Radiotherapy alone for medically inoperable stage I non-small-cell lung cancer: the Duke experience.* International Journal of Radiation Oncology, Biology, Physics, 1998. 40(1): p. 149-54.
52. Martel, M.K., et al., *Volume and dose parameters for survival of non-small cell lung cancer patients.* Radiotherapy & Oncology, 1997. 44(1): p. 23-9.
53. Kupelian, P.A., R. Komaki, and P. Allen, *Prognostic factors in the treatment of node-negative nonsmall cell lung carcinoma with radiotherapy alone.* International Journal of Radiation Oncology, Biology, Physics, 1996. 36(3): p. 607-13.
54. Hayakawa, K., et al., *Impact of tumor extent and location on treatment outcome in patients with stage III non-small cell lung cancer treated with radiation therapy.* Japanese Journal of Clinical Oncology, 1996. 26(4): p. 221-8.
55. Koukourakis, M., et al., *Radiotherapy alone for non-small cell lung carcinoma. Five-year disease-free survival and patterns of failure.* Acta Oncologica, 1995. 34(4): p. 525-30.
56. Jeremic, B. and Y. Shibamoto, *Pre-treatment prognostic factors in patients with stage III non-small cell lung cancer treated with hyperfractionated radiation therapy with or without concurrent chemotherapy.* Lung Cancer, 1995. 13(1): p. 21-30.
57. Furuta, M., et al., *Clinical implication of symptoms in patients with non-small cell lung cancer treated with definitive radiation therapy.* Lung Cancer, 1995. 13(3): p. 275-83.
58. Iaffaioli, R., et al., *Hyperfractionated split-course thoracic radiation-therapy plus chemotherapy in locally advanced nonsmall cell lung-cancer.* International Journal of Oncology, 1994. 4(3): p. 577-82.
59. Kaskowitz, L., et al., *Radiation therapy alone for stage I non-small cell lung cancer.* International Journal of Radiation Oncology, Biology, Physics, 1993. 27(3): p. 517-23.
60. Hazuka, M.B., et al., *Results of high-dose thoracic irradiation incorporating beam's eye view display in non-small cell lung cancer: a retrospective multivariate analysis.* International Journal of Radiation Oncology, Biology, Physics, 1993. 27(2): p. 273-84.
61. Dosoretz, D.E., et al., *Local control in medically inoperable lung cancer: an analysis of its importance in outcome and factors determining the probability of tumor eradication.* International Journal of Radiation Oncology, Biology, Physics, 1993. 27(3): p. 507-16.
62. Wigren, T., P. Kellokumpu-Lehtinen, and A. Ojala, *Radical radiotherapy of inoperable non-small cell lung cancer. Irradiation techniques and tumor characteristics in relation to local control and survival.* Acta Oncologica, 1992. 31(5): p. 555-61.
63. Herbert, S.H., et al., *Comparison of outcome between clinically staged, unresected superior sulcus tumors and other stage III non-small cell lung carcinomas treated with radiation therapy alone.* Cancer, 1992. 69(2): p. 363-9.
64. Herbert, S.H., et al., *Adverse influence of younger age on outcome in patients with non-small cell lung carcinoma (NSCLC) treated with radiation therapy (RT) alone.* International Journal of Radiation Oncology, Biology, Physics, 1992. 24(1): p. 37-42.
65. Sandler, H.M., W.J. Curran, Jr., and A.T. Turrisi, 3rd, *The influence of tumor size and pre-treatment staging on outcome following radiation therapy alone for stage I non-small cell lung cancer.* International Journal of Radiation Oncology, Biology, Physics, 1990. 19(1): p. 9-13.
66. Baumann, P., et al., *Outcome in a prospective phase II trial of medically inoperable stage I non-small-cell lung cancer patients treated with stereotactic body radiotherapy.* Journal of Clinical Oncology, 2009. 27(20): p. 3290-6.
67. Gao, Y., et al., *Analysis of the characteristics and prognosis of advanced non-small-cell lung cancer in older patients.* Patient preference & adherence, 2015. 9: p. 1189-94.
68. Pan, Y., et al., *Acute esophagitis for patients with local-regional advanced non small cell lung cancer treated with concurrent chemoradiotherapy.* Radiotherapy & Oncology, 2016. 118(3): p. 465-70.
69. Agarwal, J.P., et al., *Optimizing treatment and analysis of prognostic factors for locally advanced nonsmall cell lung cancer in resource-limited population.* Indian Journal of Cancer, 2016. 53(1): p. 96-101.
70. Pu, X., et al., *Inflammation-related genetic variants predict toxicity following definitive radiotherapy for lung cancer.* Clinical Pharmacology & Therapeutics, 2014. 96(5): p. 609-15.
71. Shultz, D.B., et al., *Imaging features associated with disease progression after stereotactic ablative radiotherapy for stage I non-small-cell lung cancer.* Clinical Lung Cancer, 2014. 15(4): p. 294-301.e3.
72. Smith, S.L., et al., *Inoperable early stage non-small cell lung cancer: comorbidity, patterns of care and survival.* Lung Cancer, 2011. 72(1): p. 39-44.
73. Soliman, M., et al., *GTV differentially impacts locoregional control of non-small cell lung cancer (NSCLC) after different fractionation schedules: subgroup analysis of the prospective randomized CHARTWEL trial.* Radiotherapy & Oncology, 2013. 106(3): p. 299-304.
74. Stanic, S., et al., *No clinically significant changes in pulmonary function following stereotactic body radiation therapy for early- stage peripheral non-small cell lung cancer: an analysis of RTOG 0236.* International Journal of Radiation Oncology, Biology, Physics, 2014. 88(5): p. 1092-9.
75. Unal, D., et al., *ABO blood groups are not associated with treatment response and prognosis in patients with local advanced non- small cell lung cancer.* Asian Pacific Journal of Cancer Prevention: Apjcp, 2013. 14(6): p. 3945-8.
76. Barriger, R.B., et al., *A dose-volume analysis of radiation pneumonitis in non-small cell lung cancer patients treated with stereotactic body radiation therapy.* International Journal of Radiation Oncology, Biology, Physics, 2012. 82(1): p. 457-62.
77. Horinouchi, H., et al., *Brain metastases after definitive concurrent chemoradiotherapy in patients with stage III lung adenocarcinoma: carcinoembryonic antigen as a potential predictive factor.* Cancer Science, 2012. 103(4): p. 756-9.
78. Ji, Z., et al., *Risk factors for brain metastases in locally advanced non-small cell lung cancer with definitive chest radiation.* International Journal of Radiation Oncology, Biology, Physics, 2014. 89(2): p. 330-7.
79. Li, R., et al., *MiRNA-Related Genetic Variations Associated with Radiotherapy-Induced Toxicities in Patients with Locally Advanced Non-Small Cell Lung Cancer.* PLoS ONE [Electronic Resource], 2016. 11(3): p. e0150467.
80. Machtay, M., et al., *Higher biologically effective dose of radiotherapy is associated with improved outcomes for locally advanced non-small cell lung carcinoma treated with chemoradiation: an analysis of the Radiation Therapy Oncology Group.* International Journal of Radiation Oncology, Biology, Physics, 2012. 82(1): p. 425-34.
81. Machtay, M., et al., *Defining local-regional control and its importance in locally advanced non-small cell lung carcinoma.* Journal of Thoracic Oncology: Official Publication of the International Association for the Study of Lung Cancer, 2012. 7(4): p. 716-22.
82. Makita, C., et al., *High-dose proton beam therapy for stage I non-small cell lung cancer: Clinical outcomes and prognostic factors.* Acta Oncologica, 2015. 54(3): p. 307-14.
83. Ohno, Y., et al., *Diffusion-weighted MRI versus 18F-FDG PET/CT: performance as predictors of tumor treatment response and patient survival in patients with non-small cell lung cancer receiving chemoradiotherapy.* AJR. American Journal of Roentgenology, 2012. 198(1): p. 75-82.
84. Palma, D.A., et al., *Predicting radiation pneumonitis after chemoradiation therapy for lung cancer: an international individual patient data meta-analysis.* International Journal of Radiation Oncology, Biology, Physics, 2013. 85(2): p. 444-50.
85. Salama, J.K., et al., *Pulmonary toxicity in Stage III non-small cell lung cancer patients treated with high-dose (74 Gy) 3-dimensional conformal thoracic radiotherapy and concurrent chemotherapy following induction chemotherapy: a secondary analysis of Cancer and Leukemia Group B (CALGB) trial 30105.* International Journal of Radiation Oncology, Biology, Physics, 2011. 81(4): p. e269-74.
86. Sanders, K.J., et al., *Early Weight Loss during Chemoradiotherapy Has a Detrimental Impact on Outcome in NSCLC.* Journal of Thoracic Oncology: Official Publication of the International Association for the Study of Lung Cancer, 2016. 11(6): p. 873-9.
87. Sekine, I., et al., *Gender difference in treatment outcomes in patients with stage III non-small cell lung cancer receiving concurrent chemoradiotherapy.* Japanese Journal of Clinical Oncology, 2009. 39(11): p. 707-12.
88. Shirvani, S.M., et al., *Intensity modulated radiotherapy for stage III non-small cell lung cancer in the United States: predictors of use and association with toxicities.* Lung Cancer, 2013. 82(2): p. 252-9.
89. Tang, C., et al., *Association between white blood cell count following radiation therapy with radiation pneumonitis in non-small cell lung cancer.* International Journal of Radiation Oncology, Biology, Physics, 2014. 88(2): p. 319-25.
90. Ueki, N., et al., *Impact of pretreatment interstitial lung disease on radiation pneumonitis and survival after stereotactic body radiation therapy for lung cancer.* Journal of Thoracic Oncology: Official Publication of the International Association for the Study of Lung Cancer, 2015. 10(1): p. 116-25.
91. Uyterlinde, W., et al., *Prognostic parameters for acute esophagus toxicity in intensity modulated radiotherapy and concurrent chemotherapy for locally advanced non-small cell lung cancer.* Radiotherapy & Oncology, 2013. 107(3): p. 392-7.
92. Wang, H., et al., *Do angiotensin-converting enzyme inhibitors reduce the risk of symptomatic radiation pneumonitis in patients with non-small cell lung cancer after definitive radiation therapy? Analysis of a single-institution database.* International Journal of Radiation Oncology, Biology, Physics, 2013. 87(5): p. 1071-7.
93. Wang, J., et al., *Poor baseline pulmonary function may not increase the risk of radiation-induced lung toxicity.* International Journal of Radiation Oncology, Biology, Physics, 2013. 85(3): p. 798-804.
94. Wijsman, R., et al., *Multivariable normal-tissue complication modeling of acute esophageal toxicity in advanced stage non-small cell lung cancer patients treated with intensity-modulated (chemo-)radiotherapy.* Radiotherapy & Oncology, 2015. 117(1): p. 49-54.
95. Yin, M., et al., *Functional polymorphisms of base excision repair genes XRCC1 and APEX1 predict risk of radiation pneumonitis in patients with non-small cell lung cancer treated with definitive radiation therapy.* International Journal of Radiation Oncology, Biology, Physics, 2011. 81(3): p. e67-73.
96. Yuan, S.T., et al., *Genetic variations in TGFbeta1, tPA, and ACE and radiation-induced thoracic toxicities in patients with non-small-cell lung cancer.* Journal of Thoracic Oncology: Official Publication of the International Association for the Study of Lung Cancer, 2013. 8(2): p. 208-13.
97. Yuan, X., et al., *Single nucleotide polymorphism at rs1982073:T869C of the TGFbeta 1 gene is associated with the risk of radiation pneumonitis in patients with non-small-cell lung cancer treated with definitive radiotherapy.* Journal of Clinical Oncology, 2009. 27(20): p. 3370-8.
98. Zehentmayr, F., et al., *Normal tissue complication models for clinically relevant acute esophagitis (> grade 2) in patients treated with dose differentiated accelerated radiotherapy (DART-bid).* Radiation Oncology, 2015. 10: p. 121.
99. Abelson, J.A., et al., *Metabolic imaging metrics correlate with survival in early stage lung cancer treated with stereotactic ablative radiotherapy.* Lung Cancer, 2012. 78(3): p. 219-24.
100. Alexander, B.M., et al., *Tumor volume is a prognostic factor in non-small-cell lung cancer treated with chemoradiotherapy.* International Journal of Radiation Oncology, Biology, Physics, 2011. 79(5): p. 1381-7.
101. Allibhai, Z., et al., *The impact of tumor size on outcomes after stereotactic body radiation therapy for medically inoperable early-stage non-small cell lung cancer.* International Journal of Radiation Oncology, Biology, Physics, 2013. 87(5): p. 1064-70.
102. Andratschke, N., et al., *Stereotactic radiotherapy of histologically proven inoperable stage I non-small cell lung cancer: patterns of failure.* Radiotherapy & Oncology, 2011. 101(2): p. 245-9.
103. Arslan, D., et al., *Prognostic factors in clinical stage T4N2 locally advanced non-small cell lung cancer.* Journal of B.U.On., 2015. 20(2): p. 573-9.
104. Atallah, S., et al., *Impact of pretreatment tumor growth rate on outcome of early-stage lung cancer treated with stereotactic body radiation therapy.* International Journal of Radiation Oncology, Biology, Physics, 2014. 89(3): p. 532-8.
105. Bahig, H., et al., *Excellent Cancer Outcomes Following Patient-adapted Robotic Lung SBRT But a Case for Caution in Idiopathic Pulmonary Fibrosis.* Technology in Cancer Research & Treatment, 2015. 14(6): p. 667-76.
106. Bi, N., et al., *Cyclooxygenase-2 genetic variants are associated with survival in unresectable locally advanced non-small cell lung cancer.* Clinical Cancer Research, 2010. 16(8): p. 2383-90.
107. Briere, T.M., et al., *Lung Size and the Risk of Radiation Pneumonitis.* International Journal of Radiation Oncology, Biology, Physics, 2016. 94(2): p. 377-84.
108. Bush, D.A., et al., *High-dose hypofractionated proton beam radiation therapy is safe and effective for central and peripheral early-stage non-small cell lung cancer: results of a 12-year experience at Loma Linda University Medical Center.* International Journal of Radiation Oncology, Biology, Physics, 2013. 86(5): p. 964-8.
109. Butkiewicz, D., et al., *The VEGFR2, COX-2 and MMP-2 polymorphisms are associated with clinical outcome of patients with inoperable non-small cell lung cancer.* International Journal of Cancer, 2015. 137(10): p. 2332-42.
110. Caglar, H.B., M. Othus, and A.M. Allen, *Esophagus in-field: a new predictor for esophagitis.* Radiotherapy & Oncology, 2010. 97(1): p. 48-53.
111. Cannon, N.A., et al., *Neutrophil-lymphocyte and platelet-lymphocyte ratios as prognostic factors after stereotactic radiation therapy for early-stage non-small-cell lung cancer.* Journal of Thoracic Oncology: Official Publication of the International Association for the Study of Lung Cancer, 2015. 10(2): p. 280-5.
112. Chang, J.Y., et al., *Clinical outcome and predictors of survival and pneumonitis after stereotactic ablative radiotherapy for stage I non-small cell lung cancer.* Radiation Oncology, 2012. 7: p. 152.
113. Chen, C., et al., *Severe late esophagus toxicity in NSCLC patients treated with IMRT and concurrent chemotherapy.* Radiotherapy & Oncology, 2013. 108(2): p. 337-41.
114. Cheung, P., et al., *Phase II study of accelerated hypofractionated three-dimensional conformal radiotherapy for stage T1-3 N0 M0 non-small cell lung cancer: NCIC CTG BR.25.[Erratum appears in J Natl Cancer Inst. 2015 Jan;107(1): dju430 doi:10.1093/jnci/dju430].* Journal of the National Cancer Institute, 2014. 106(8).
115. Chiang, A., et al., *A comparison between accelerated hypofractionation and stereotactic ablative radiotherapy (SABR) for early-stage non-small cell lung cancer (NSCLC): Results of a propensity score-matched analysis.* Radiotherapy & Oncology, 2016. 118(3): p. 478-84.
116. Cihan, Y.B., *Do trace element levels have prognostic value in non-small cell lung cancer patients treated with chemoradiotherapy?* Journal of B.U.On., 2014. 19(3): p. 749-56.
117. Clarke, K., et al., *Stereotactic body radiotherapy (SBRT) for non-small cell lung cancer (NSCLC): is FDG-PET a predictor of outcome?* Radiotherapy & Oncology, 2012. 104(1): p. 62-6.
118. Cook, G.J., et al., *Are pretreatment 18F-FDG PET tumor textural features in non-small cell lung cancer associated with response and survival after chemoradiotherapy?* Journal of Nuclear Medicine, 2013. 54(1): p. 19-26.
119. Crvenkova, S., *Prognostic Factors and Survival in Non-Small Cell Lung Cancer Patients Treated with Chemoradiotherapy.* Open Access Macedonian Journal of Medical Sciences, 2015. 3(1): p. 75-9.
120. Crvenkova, S. and M. Pesevska, *Important prognostic factors for the long-term survival in non-small cell lung cancer patients treated with combination of chemotherapy and conformal radiotherapy.* Journal of B.U.On., 2015. 20(3): p. 775-81.
121. Cuaron, J.J., et al., *Stereotactic body radiation therapy for primary lung cancers >3 centimeters.* Journal of Thoracic Oncology: Official Publication of the International Association for the Study of Lung Cancer, 2013. 8(11): p. 1396-401.
122. Dang, J., et al., *Risk and predictors for early radiation pneumonitis in patients with stage III non-small cell lung cancer treated with concurrent or sequential chemoradiotherapy.* Radiation Oncology, 2014. 9: p. 172.
123. Fakiris, A.J., et al., *Stereotactic body radiation therapy for early-stage non-small-cell lung carcinoma: four-year results of a prospective phase II study.* International Journal of Radiation Oncology, Biology, Physics, 2009. 75(3): p. 677-82.
124. Fried, D.V., et al., *Prognostic value and reproducibility of pretreatment CT texture features in stage III non-small cell lung cancer.* International Journal of Radiation Oncology, Biology, Physics, 2014. 90(4): p. 834-42.
125. Guckenberger, M., et al., *Is there a lower limit of pretreatment pulmonary function for safe and effective stereotactic body radiotherapy for early-stage non-small cell lung cancer?* Journal of Thoracic Oncology: Official Publication of the International Association for the Study of Lung Cancer, 2012. 7(3): p. 542-51.
126. Hayashi, S., et al., *Stereotactic body radiotherapy for very elderly patients (age, greater than or equal to 85 years) with stage I non-small cell lung cancer.* Radiation Oncology, 2014. 9: p. 138.
127. He, J., et al., *Feasibility and efficacy of helical intensity-modulated radiotherapy for stage III non-small cell lung cancer in comparison with conventionally fractionated 3D-CRT.* Journal of Thoracic Disease, 2016. 8(5): p. 862-71.
128. Horinouchi, H., et al., *Candidates for Intensive Local Treatment in cIIIA-N2 Non-Small Cell Lung Cancer: Deciphering the Heterogeneity.* International Journal of Radiation Oncology, Biology, Physics, 2016. 94(1): p. 155-62.
129. Horne, Z.D., et al., *Pretreatment SUVmax predicts progression-free survival in early-stage non-small cell lung cancer treated with stereotactic body radiation therapy.* Radiation Oncology, 2014. 9: p. 41.
130. Huang, W., et al., *The early predictive value of a decrease of metabolic tumor volume in repeated (18)F-FDG PET/CT for recurrence of locally advanced non-small cell lung cancer with concurrent radiochemotherapy.* European Journal of Radiology, 2015. 84(3): p. 482-8.
131. Inoue, T., et al., *Stereotactic body radiotherapy using gated radiotherapy with real-time tumor-tracking for stage I non-small cell lung cancer.* Radiation Oncology, 2013. 8: p. 69.
132. Jalal, S.I., et al., *Updated survival and outcomes for older adults with inoperable stage III non-small-cell lung cancer treated with cisplatin, etoposide, and concurrent chest radiation with or without consolidation docetaxel: analysis of a phase III trial from the Hoosier Oncology Group (HOG) and US Oncology.* Annals of Oncology, 2012. 23(7): p. 1730-8.
133. Jenkins, P. and J. Watts, *An improved model for predicting radiation pneumonitis incorporating clinical and dosimetric variables.* International Journal of Radiation Oncology, Biology, Physics, 2011. 80(4): p. 1023-9.
134. Jeppesen, S.S., et al., *Stereotactic body radiation therapy versus conventional radiation therapy in patients with early stage non-small cell lung cancer: an updated retrospective study on local failure and survival rates.* Acta Oncologica, 2013. 52(7): p. 1552-8.
135. Jeremic, B., B. Milicic, and S. Milisavljevic, *Clinical prognostic factors in patients with locally advanced (stage III) nonsmall cell lung cancer treated with hyperfractionated radiation therapy with and without concurrent chemotherapy: single-Institution Experience in 600 Patients.* Cancer, 2011. 117(13): p. 2995-3003.
136. Jeremic, B., B. Milicic, and S. Milisavljevic, *Toxicity of concurrent hyperfractionated radiation therapy and chemotherapy in locally advanced (stage III) non-small cell lung cancer (NSCLC): single institution experience in 600 patients.* Clinical & Translational Oncology: Official Publication of the Federation of Spanish Oncology Societes & of the National Cancer Institute of Mexico, 2012. 14(8): p. 613-8.
137. Kanemoto, A., et al., *Outcomes and prognostic factors for recurrence after high-dose proton beam therapy for centrally and peripherally located stage I non--small-cell lung cancer.* Clinical Lung Cancer, 2014. 15(2): p. e7-12.
138. Kang, H.C., et al., *Fluorodeoxyglucose positron-emission tomography ratio in non-small cell lung cancer patients treated with definitive radiotherapy.* Radiation Oncology Journal, 2013. 31(3): p. 111-7.
139. Kim, Y.H., et al., *Predictive factors for survival and correlation to toxicity in advanced Stage III non-small cell lung cancer patients with concurrent chemoradiation.* Japanese Journal of Clinical Oncology, 2016. 46(2): p. 144-51.
140. Kishi, T., et al., *Pretreatment Modified Glasgow Prognostic Score Predicts Clinical Outcomes After Stereotactic Body Radiation Therapy for Early-Stage Non-Small Cell Lung Cancer.* International Journal of Radiation Oncology, Biology, Physics, 2015. 92(3): p. 619-26.
141. Kohutek, Z.A., et al., *FDG-PET maximum standardized uptake value is prognostic for recurrence and survival after stereotactic body radiotherapy for non-small cell lung cancer.* Lung Cancer, 2015. 89(2): p. 115-20.
142. Kolodziejczyk, M., et al., *[Outcome of three-dimensional conformal radiotherapy for early stage non-small cell lung cancer patients who met or not inclusion criteria for stereotactic-body radiation therapy].* Pneumonologia i Alergologia Polska, 2011. 79(5): p. 326-36.
143. Komaki, R., et al., *EGFR expression and survival in patients given cetuximab and chemoradiation for stage III non-small cell lung cancer: a secondary analysis of RTOG 0324.* Radiotherapy & Oncology, 2014. 112(1): p. 30-6.
144. Koo, T.R., et al., *The effect of tumor volume and its change on survival in stage III non-small cell lung cancer treated with definitive concurrent chemoradiotherapy.* Radiation Oncology, 2014. 9: p. 283.
145. Kurtul, N., et al., *Prognostic value of SPARC expression in unresectable NSCLC treated with concurrent chemoradiotherapy.* Asian Pacific Journal of Cancer Prevention: Apjcp, 2014. 15(20): p. 8911-6.
146. Lee, J.H., et al., *Influence of Comorbidities on the Efficacy of Radiotherapy with or without Chemotherapy in Elderly Stage III Non-small Cell Lung Cancer Patients.* Cancer Research & Treatment, 2012. 44(4): p. 242-50.
147. Lee, S., et al., *Bayesian network ensemble as a multivariate strategy to predict radiation pneumonitis risk.* Medical Physics, 2015. 42(5): p. 2421-30.
148. Liao, Z.X., et al., *Influence of technologic advances on outcomes in patients with unresectable, locally advanced non-small-cell lung cancer receiving concomitant chemoradiotherapy.* International Journal of Radiation Oncology, Biology, Physics, 2010. 76(3): p. 775-81.
149. Lucas, J.T., Jr., et al., *Comparison of accelerated hypofractionation and stereotactic body radiotherapy for Stage 1 and node negative Stage 2 non-small cell lung cancer (NSCLC).* Lung Cancer, 2014. 85(1): p. 59-65.
150. Mak, R.H., et al., *Outcomes by tumor histology and KRAS mutation status after lung stereotactic body radiation therapy for early-stage non-small-cell lung cancer.* Clinical Lung Cancer, 2015. 16(1): p. 24-32.
151. Marwaha, G., et al., *Lung stereotactic body radiation therapy: regional nodal failure is not predicted by tumor size.* Journal of Thoracic Oncology: Official Publication of the International Association for the Study of Lung Cancer, 2014. 9(11): p. 1693-7.
152. Massabeau, C., et al., *The prognostic significance of lymphovascular invasion on biopsy specimens in lung cancer treated with definitive chemoradiotherapy.* Clinical Lung Cancer, 2012. 13(1): p. 59-67.
153. Massabeau, C., et al., *Basic fibroblast growth factor-2/beta3 integrin expression profile: signature of local progression after chemoradiotherapy for patients with locally advanced non-small-cell lung cancer.* International Journal of Radiation Oncology, Biology, Physics, 2009. 75(3): p. 696-702.
154. Matsuo, Y., et al., *Prognostic factors in stereotactic body radiotherapy for non-small-cell lung cancer.* International Journal of Radiation Oncology, Biology, Physics, 2011. 79(4): p. 1104-11.
155. Milano, M.T., et al., *Definitive radiotherapy for stage I nonsmall cell lung cancer: a population-based study of survival.* Cancer, 2012. 118(22): p. 5572-9.
156. Nair, V.J., et al., *Pretreatment [18F]-fluoro-2-deoxy-glucose positron emission tomography maximum standardized uptake value as predictor of distant metastasis in early-stage non-small cell lung cancer treated with definitive radiation therapy: rethinking the role of positron emission tomography in personalizing treatment based on risk status.* International Journal of Radiation Oncology, Biology, Physics, 2014. 88(2): p. 312-8.
157. Nakayama, H., et al., *High-dose conformal radiotherapy for patients with stage III non-small-cell lung carcinoma.* International Journal of Radiation Oncology, Biology, Physics, 2010. 78(3): p. 645-50.
158. Oh, D., et al., *Hypofractionated three-dimensional conformal radiation therapy alone for centrally located cT1-3N0 non-small-cell lung cancer.* Journal of Thoracic Oncology: Official Publication of the International Association for the Study of Lung Cancer, 2013. 8(5): p. 624-9.
159. Oh, D., et al., *Prediction of radiation pneumonitis following high-dose thoracic radiation therapy by 3 Gy/fraction for non-small cell lung cancer: analysis of clinical and dosimetric factors.* Japanese Journal of Clinical Oncology, 2009. 39(3): p. 151-7.
160. Oh, J.H., et al., *A Bayesian network approach for modeling local failure in lung cancer.* Physics in Medicine & Biology, 2011. 56(6): p. 1635-51.
161. Ohri, N., et al., *Pretreatment FDG-PET metrics in stage III non-small cell lung cancer: ACRIN 6668/RTOG 0235.* Journal of the National Cancer Institute, 2015. 107(4).
162. Olsen, J.R., et al., *Dose-response for stereotactic body radiotherapy in early-stage non-small-cell lung cancer.* International Journal of Radiation Oncology, Biology, Physics, 2011. 81(4): p. e299-303.
163. Palma, D.A., et al., *Stage I non-small cell lung cancer (NSCLC) in patients aged 75 years and older: does age determine survival after radical treatment?* Journal of Thoracic Oncology: Official Publication of the International Association for the Study of Lung Cancer, 2010. 5(6): p. 818-24.
164. Park, H.S., et al., *Central versus Peripheral Tumor Location: Influence on Survival, Local Control, and Toxicity Following Stereotactic Body Radiotherapy for Primary Non-Small-Cell Lung Cancer.* Journal of Thoracic Oncology: Official Publication of the International Association for the Study of Lung Cancer, 2015. 10(5): p. 832-7.
165. Ricardi, U., et al., *Stereotactic Ablative Radiotherapy for stage I histologically proven non-small cell lung cancer: an Italian multicenter observational study.* Lung Cancer, 2014. 84(3): p. 248-53.
166. Satoh, Y., et al., *Value of dual time point F-18 FDG-PET/CT imaging for the evaluation of prognosis and risk factors for recurrence in patients with stage I non-small cell lung cancer treated with stereotactic body radiation therapy.* European Journal of Radiology, 2012. 81(11): p. 3530-4.
167. Satoh, Y., et al., *Volume-based parameters measured by using FDG PET/CT in patients with stage I NSCLC treated with stereotactic body radiation therapy: prognostic value.* Radiology, 2014. 270(1): p. 275-81.
168. Semrau, S., G. Klautke, and R. Fietkau, *Baseline cardiopulmonary function as an independent prognostic factor for survival of inoperable non-small-cell lung cancer after concurrent chemoradiotherapy: a single-center analysis of 161 cases.* International Journal of Radiation Oncology, Biology, Physics, 2011. 79(1): p. 96-104.
169. Shirata, Y., et al., *Prognostic factors for local control of stage I non-small cell lung cancer in stereotactic radiotherapy: a retrospective analysis.* Radiation Oncology, 2012. 7: p. 182.
170. Stenmark, M.H., et al., *Combining physical and biologic parameters to predict radiation-induced lung toxicity in patients with non-small-cell lung cancer treated with definitive radiation therapy.* International Journal of Radiation Oncology, Biology, Physics, 2012. 84(2): p. e217-22.
171. Takeda, A., et al., *Stereotactic ablative body radiation therapy for octogenarians with non-small cell lung cancer.* International Journal of Radiation Oncology, Biology, Physics, 2013. 86(2): p. 257-63.
172. Takeda, A., et al., *Maximum standardized uptake value on FDG-PET is a strong predictor of overall and disease-free survival for non-small-cell lung cancer patients after stereotactic body radiotherapy.* Journal of Thoracic Oncology: Official Publication of the International Association for the Study of Lung Cancer, 2014. 9(1): p. 65-73.
173. Takeda, A., et al., *The maximum standardized uptake value (SUVmax) on FDG-PET is a strong predictor of local recurrence for localized non-small-cell lung cancer after stereotactic body radiotherapy (SBRT).* Radiotherapy & Oncology, 2011. 101(2): p. 291-7.
174. Tsujino, K., et al., *Combined analysis of V20, VS5, pulmonary fibrosis score on baseline computed tomography, and patient age improves prediction of severe radiation pneumonitis after concurrent chemoradiotherapy for locally advanced non-small-cell lung cancer.* Journal of Thoracic Oncology: Official Publication of the International Association for the Study of Lung Cancer, 2014. 9(7): p. 983-90.
175. Tsurugai, Y., et al., *Relationship between the consolidation to maximum tumor diameter ratio and outcomes following stereotactic body radiotherapy for stage I non-small-cell lung cancer.* Lung Cancer, 2016. 92: p. 47-52.
176. Tucker, S.L., et al., *Incorporating single-nucleotide polymorphisms into the Lyman model to improve prediction of radiation pneumonitis.* International Journal of Radiation Oncology, Biology, Physics, 2013. 85(1): p. 251-7.
177. Ulger, S., et al., *High FDG uptake predicts poorer survival in locally advanced nonsmall cell lung cancer patients undergoing curative radiotherapy, independently of tumor size.* Journal of Cancer Research & Clinical Oncology, 2014. 140(3): p. 495-502.
178. Unal, D., et al., *Are neutrophil/lymphocyte and platelet/lymphocyte rates in patients with non-small cell lung cancer associated with treatment response and prognosis?* Asian Pacific Journal of Cancer Prevention: Apjcp, 2013. 14(9): p. 5237-42.
179. Vera, P., et al., *FDG PET during radiochemotherapy is predictive of outcome at 1 year in non-small-cell lung cancer patients: a prospective multicentre study (RTEP2).* European Journal of Nuclear Medicine & Molecular Imaging, 2014. 41(6): p. 1057-65.
180. Vu, C.C., et al., *Prognostic value of metabolic tumor volume and total lesion glycolysis from 18F-FDG PET/CT in patients undergoing stereotactic body radiation therapy for stage I non-small-cell lung cancer.* Nuclear Medicine Communications, 2013. 34(10): p. 959-63.
181. Wang, H.M., et al., *Improved survival outcomes with the incidental use of beta-blockers among patients with non-small-cell lung cancer treated with definitive radiation therapy.* Annals of Oncology, 2013. 24(5): p. 1312-9.
182. Xiang, Z.L., et al., *FDG uptake correlates with recurrence and survival after treatment of unresectable stage III non-small cell lung cancer with high-dose proton therapy and chemotherapy.* Radiation Oncology, 2012. 7: p. 144.
183. Yagishita, S., et al., *Impact of KRAS mutation on response and outcome of patients with stage III non-squamous non-small cell lung cancer.* Cancer Science, 2015. 106(10): p. 1402-7.
184. Yamamoto, T., et al., *Formula corrected maximal standardized uptake value in FDG-PET for partial volume effect and motion artifact is not a prognostic factor in stage I non-small cell lung cancer treated with stereotactic body radiotherapy.* Annals of Nuclear Medicine, 2015. 29(8): p. 666-73.
185. Yuan, X., et al., *TGFbeta1 Polymorphisms Predict Distant Metastasis-Free Survival in Patients with Inoperable Non-Small-Cell Lung Cancer after Definitive Radiotherapy.* PLoS ONE [Electronic Resource], 2013. 8(6): p. e65659.
186. Zhao, L., et al., *Changes of circulating transforming growth factor-beta1 level during radiation therapy are correlated with the prognosis of locally advanced non-small cell lung cancer.* Journal of Thoracic Oncology: Official Publication of the International Association for the Study of Lung Cancer, 2010. 5(4): p. 521-5.
187. Zhao, L., et al., *Elevation of plasma TGF-beta1 during radiation therapy predicts radiation-induced lung toxicity in patients with non-small-cell lung cancer: a combined analysis from Beijing and Michigan.* International Journal of Radiation Oncology, Biology, Physics, 2009. 74(5): p. 1385-90.
188. Fromm, S., et al., *3D-conformal radiotherapy for inoperable non-small-cell lung cancer - A single centre experience.* Radiology and Oncology, 2007. 41: p. 133-143.
189. Uitterhoeve, A.L., et al., *Accelerated high-dose radiotherapy alone or combined with either concomitant or sequential chemotherapy; treatments of choice in patients with Non-Small Cell Lung Cancer.* Radiation Oncology, 2007. 2: p. 27.
190. Cox, J.D., et al., *Addition of chemotherapy to radiation therapy alters failure patterns by cell type within non-small cell carcinoma of lung (NSCCL): Analysis of radiation therapy oncology group (RTOG) trials.* International Journal of Radiation Oncology Biology Physics, 1999. 43: p. 505-509.
191. Germain, F., et al., *Brain metastasis is an early manifestation of distant failure in stage III nonsmall cell lung cancer patients treated with radical chemoradiation therapy.* American Journal of Clinical Oncology: Cancer Clinical Trials, 2008. 31: p. 561-566.
192. Sun, Z., et al., *Clinical analysis of concurrent chemoradiotherapy in 83 patients with locally advanced non-small cell lung cancer.* Chinese-German Journal of Clinical Oncology, 2012. 11: p. 1-5.
193. Yu, X., et al., *Clinical significance of serum soluble death receptor 5 concentration in locally advanced non-small cell lung cancer patients.* Oncology Letters, 2014. 8: p. 1333-1339.
194. Pan, D., et al., *Clinical study on gefitinib combined with gamma-ray stereotactic body radiation therapy as the first-line treatment regimen for senile patients with adenocarcinoma of the lung (final results of JLY20080085).* Molecular and Clinical Oncology, 2013. 1: p. 711-715.
195. Dang, J., et al., *Comparison of risk and predictors for early radiation pneumonitis in patients with locally advanced non-small cell lung cancer treated with radiotherapy with or without surgery.* Lung Cancer, 2014. 86: p. 329-333.
196. Boudaoud, K., et al., *Concurrent cisplatin, etoposide and chest radiotherapy in locally advanced non small cell lung carcinoma: Survival and prognostic factors in the east of Algeria.* International Journal of Pharmaceutical Sciences Review and Research, 2016. 37: p. 238-243.
197. Zatloukal, P., et al., *Concurrent versus sequential chemoradiotherapy with cisplatin and vinorelbine in locally advanced non-small cell lung cancer: A randomized study.* Lung Cancer, 2004. 46: p. 87-98.
198. Saunders, M., et al., *Continuous, hyperfractionated, accelerated radiotherapy (CHART) versus conventional radiotherapy in non-small cell lung cancer: mature data from the randomised multicentre trial. CHART Steering committee.* Radiotherapy & Oncology, 1999. 52: p. 137-48.
199. Hayakawa, K., et al., *Definitive radiation therapy for medically inoperable patients with stage I and II non-small cell lung cancer.* Radiation Oncology Investigations, 1996. 4: p. 165-170.
200. Hendriks, L.E.L., et al., *Development of symptomatic brain metastases after chemoradiotherapy for stage III non-small cell lung cancer: Does the type of chemotherapy regimen matter?* Lung Cancer, 2016. 101: p. 68-75.
201. Kestin, L., et al., *Dose-response relationship with clinical outcome for lung stereotactic body radiotherapy (SBRT) delivered via online image guidance.* Radiotherapy and Oncology, 2014. 110: p. 499-504.
202. De Ruysscher, D., et al., *Dyspnea evolution after high-dose radiotherapy in patients with non-small cell lung cancer.* Radiotherapy and Oncology, 2009. 91: p. 353-359.
203. Wang, L., et al., *An East Asian subgroup analysis of PROCLAIM, a phase III trial of pemetrexed and cisplatin or etoposide and cisplatin plus thoracic radiation therapy followed by consolidation chemotherapy in locally advanced nonsquamous non-small cell lung cancer.* Asia-Pacific Journal of Clinical Oncology, 2016. 12: p. 380-387.
204. Afsar, C.U., et al., *The effect of chemoradiotherapy on survival in locally advanced unresectable non-small cell lung cancer patients: Experience from the southeast region of Turkey.* 2015. 31: p. 883-888.
205. Tang, C., et al., *Effects of Chemotherapy Regimen and Radiation Modality on Hematologic Toxicities in Patients Receiving Definitive Platinum-based Doublet Chemoradiation for Non-Small Cell Lung Cancer.* Am J Clin Oncol, 2015.
206. Wang, T., et al., *Efficacy and safety of S-1 (tegafur, gimeracil, and oteracil potassium) concurrent with 3-dimensional conformal radiotherapy for newly diagnosed squamous cell carcinoma of the lung in elderly patients.* Cancer/Radiotherapie, 2016. 20: p. 181-186.
207. Liang, X., et al., *Efficacy of the smaller target volume for stage III non-small cell lung cancer treated with intensity-modulated radiotherapy.* Molecular and Clinical Oncology, 2015. 3: p. 1172-1176.
208. Tanaka, K., et al., *EGFR Mutation Impact on Definitive Concurrent Chemoradiation Therapy for Inoperable Stage III Adenocarcinoma.* Journal of Thoracic Oncology, 2015. 10: p. 1720-1725.
209. Ishihara, M., et al., *Evaluation of concurrent chemoradiotherapy for locally advanced NSCLC according to EGFR mutation status.* Oncology Letters, 2017. 14: p. 885-890.
210. Gouders, D., et al., *Exclusive radiotherapy for non small cell lung cancer. A retrospective multicentric study.* Reports of Practical Oncology and Radiotherapy, 2003. 8: p. 7-14.
211. Haseltine, J.M., et al., *Fatal complications after stereotactic body radiation therapy for central lung tumors abutting the proximal bronchial tree.* Practical Radiation Oncology, 2016. 6: p. e27-e33.
212. Martinez, E., et al., *Feasibility, tolerability, and efficacy of the concurrent addition of erlotinib to thoracic radiotherapy in locally advanced unresectable non-small-cell lung cancer: A phase II trial.* OncoTargets and Therapy, 2016. 9: p. 1057-1066.
213. Uyterlinde, W., et al., *Fractures of thoracic vertebrae in patients with locally advanced non-small cell lung carcinoma treated with intensity modulated radiotherapy.* Radiotherapy and Oncology, 2016. 118: p. 437-441.
214. Jeremic, B. and B. Milicic, *From conventionally fractionated radiation therapy to hyperfractionated radiation therapy alone and with concurrent chemotherapy in patients with early-stage nonsmall cell lung cancer.* Cancer, 2008. 112: p. 876-884.
215. Wang, D., et al., *Functional dose-volume histograms for predicting radiation pneumonitis in locally advanced non-small cell lung cancer treated with late-course accelerated hyperfractionated radiotherapy.* Exp Ther Med, 2012. 2: p. 1017-1022.
216. Woody, N.M., et al., *A Histologic Basis for the Efficacy of SBRT to the lung.* Journal of Thoracic Oncology, 2017. 12: p. 510-519.
217. Leeman, J.E., et al., *Histologic Subtype in Core Lung Biopsies of Early-Stage Lung Adenocarcinoma is a Prognostic Factor for Treatment Response and Failure Patterns After Stereotactic Body Radiation Therapy.* International Journal of Radiation Oncology Biology Physics, 2017. 97: p. 138-145.
218. Hayashi, S., H. Tanaka, and H. Hoshi, *Imaging characteristics of local recurrences after stereotactic body radiation therapy for stage I non-small cell lung cancer: Evaluation of mass-like fibrosis.* Thorac Cancer, 2015. 6: p. 186-93.
219. Li, Q., et al., *Imaging features from pretreatment CT scans are associated with clinical outcomes in nonsmall-cell lung cancer patients treated with stereotactic body radiotherapy.* Med Phys, 2017.
220. Yang, K., et al., *Improved local control without elective nodal radiotherapy in patients with unresectable NSCLC treated by 3D-CRT.* Front Med China, 2007. 1: p. 381-5.
221. Fischer-Valuck, B.W., et al., *Influence of patient characteristics on survival following treatment with helical stereotactic body radiotherapy (SBRT) in stage I non-small-cell lung cancer.* Thoracic Cancer, 2013. 4: p. 27-34.
222. Komiya, T., et al., *Infrequent chemoradiation-induced acute esophagitis in the Asian population: A meta-analysis of published clinical trials for unresectable stage III non-small cell lung cancer.* Thoracic Cancer, 2014. 5: p. 565-569.
223. Wang, J., et al., *Intensity-Modulated Radiation Therapy May Improve Local-Regional Tumor Control for Locally Advanced Non-Small Cell Lung Cancer Compared With Three-Dimensional Conformal Radiation Therapy.* Oncologist, 2016. 21: p. 1530-1537.
224. Agrawal, S., et al., *Ipsilateral lung dose volume parameters predict radiation pneumonitis in addition to classical dose volume parameters in locally advanced NSCLC treated with combined modality therapy.* South Asian J Cancer, 2014. 3: p. 13-5.
225. Shien, K., et al., *Lower lobe origin is a poor prognostic factor in locally advanced non-small-cell lung cancer patients treated with induction chemoradiotherapy.* Mol Clin Oncol, 2015. 3: p. 706-712.
226. Agrawal, V., et al., *Lymph node volume predicts survival but not nodal clearance in Stage IIIA-IIIB NSCLC.* PLoS ONE, 2017. 12 (4) (no pagination).
227. Kishida, Y., et al., *Myelosuppression induced by concurrent hemoradiotherapy as a prognostic factor for patients with locally advanced non-small cell lung cancer.* Oncology Letters, 2011. 2: p. 949-955.
228. Petrovic, M., et al., *Neuroendocrine Markers-Useful Predictors of Therapeutic Responses in Non-resectable Non-small Cell Lung Cancer.* Laboratory Medicine, 2012. 43: p. 6-10.
229. Lee, Y.H., et al., *Neutrophil-lymphocyte ratio and a dosimetric factor for predicting symptomatic radiation pneumonitis in non-small-cell lung cancer patients treated with concurrent chemoradiotherapy.* Clin Respir J, 2017.
230. Fondevilla Soler, A., et al., *Outcome and toxicity of intensity modulated radiotherapy with simultaneous integrated boost in locally advanced non-small cell lung cancer patients.* Clin Transl Oncol, 2017.
231. Junker, K., et al., *p53 Tumour-suppressor gene in non-small-cell lung cancer with neoadjuvant therapy.* Journal of Cancer Research and Clinical Oncology, 2000. 126: p. 238-245.
232. Shumway, D., et al., *Pathologic response rates following definitive dose image-guided chemoradiotherapy and resection for locally advanced non-small cell lung cancer.* Lung Cancer, 2011. 74: p. 446-450.
233. Lin, H., et al., *Phase 3 randomized low-dose paclitaxel chemoradiotherapy study for locally advanced non-small cell lung cancer.* Frontiers in Oncology, 2016. 6 (DEC) (no pagination).
234. Vera, P., et al., *Phase II Study of a Radiotherapy Total Dose Increase in Hypoxic Lesions Identified by 18F-Misonidazole PET/CT in Patients with Non-Small Cell Lung Carcinoma (RTEP5 Study).* J Nucl Med, 2017. 58: p. 1045-1053.
235. Belani, C.P., et al., *Phase III study of the Eastern Cooperative Oncology Group (ECOG 2597): Induction chemotherapy followed by either standard thoracic radiotherapy or hyperfractionated accelerated radiotherapy for patients with unresectable stage IIIA and B non-small-cell lung cancer.* Journal of Clinical Oncology, 2005. 23: p. 3760-3767.
236. Soliman, M., *Predictive factors of radiation-induced lung toxicity in lung cancer patients: A retrospective study.* Middle East Journal of Cancer, 2016. 7: p. 137-143.
237. Soliman, M., *Predictors of early radiation induced esophageal toxicity in radiotherapy of locally advanced non-small cell lung cancer.* Middle East Journal of Cancer, 2017. 8: p. 135-141.
238. Senan, S., et al., *PROCLAIM: Randomized Phase III trial of pemetrexed-cisplatin or etoposide-cisplatin plus thoracic radiation therapy followed by consolidation chemotherapy in locally advanced nonsquamous non-small-cell lung cancer.* Journal of Clinical Oncology, 2016. 34: p. 953-962.
239. Hayashi, K., et al., *Prognostic analysis of radiation pneumonitis: carbon-ion radiotherapy in patients with locally advanced lung cancer.* Radiat Oncol, 2017. 12: p. 91.
240. Hong, J., et al., *Prognostic Factors as a Function of Disease-free Interval After Definitive (Chemo)radiation for Non-Small Cell Lung Cancer Using Conditional Survival Analysis.* Am J Clin Oncol, 2015.
241. Deek, M.P., et al., *Prognostic Impact of Missed Chemotherapy Doses During Chemoradiation Therapy for Non-Small Cell Lung Cancer.* American Journal of Clinical Oncology: Cancer Clinical Trials., 2016. 17.
242. Agrawal, V., et al., *Radiologic-pathologic correlation of response to chemoradiation in resectable locally advanced NSCLC.* Lung Cancer, 2016. 102: p. 1-8.
243. Jeremic, B., B. Milicic, and S. Milisavljevic, *Radiotherapy alone versus radiochemotherapy in patients with stage IIIA adenocarcinoma (ADC) of the lung.* Clinical and Translational Oncology, 2013. 15: p. 747-753.
244. Goldsmith, B., J. Cesaretti, and J.P. Wisnivesky, *Radiotherapy Planning Complexity and Survival after Treatment of Advanced Stage Lung Cancer in the Elderly.* Cancer, 2009. 115: p. 4865-4873.
245. Senan, S., et al., *A randomized phase II study comparing induction or consolidation chemotherapy with cisplatin-docetaxel, plus radical concurrent chemoradiotherapy with cisplatin-docetaxel, in patients with unresectable locally advanced non-small-cell lung cancer.* Annals of Oncology, 2011. 22: p. 553-558.
246. Wang, L., et al., *Randomized phase II study of concurrent cisplatin/etoposide or paclitaxel/carboplatin and thoracic radiotherapy in patients with stage III non-small cell lung cancer.* Lung Cancer, 2012. 77: p. 89-96.
247. Gouda, Y.S., et al., *Randomized study of concurrent carboplatin, paclitaxel, and radiotherapy with or without prior induction chemotherapy in patients with locally advanced non-small cell lung cancer.* Journal of Egyptian National Cancer Institute, 2006. 18: p. 73-81.
248. Jeremic, B., et al., *Randomized trial of hyperfractionated radiation therapy with or without concurrent chemotherapy for stage III non-small-cell lung cancer.* Journal of Clinical Oncology, 1995. 13: p. 452-458.
249. Dillman, R.O., et al., *A randomized trial of induction chemotherapy plus high-dose radiation versus radiation alone in Stage III non-small-cell lung cancer.* New England Journal of Medicine, 1990. 323: p. 940-945.
250. Byhardt, R.W., et al., *Response, toxicity, failure patterns, and survival in five radiation therapy oncology group (RTOG) trials of sequential and/or concurrent chemotherapy and radiotherapy for locally advanced non-small-cell carcinoma of the lung.* International Journal of Radiation Oncology Biology Physics, 1998. 42: p. 469-478.
251. Kepka, L., K. Bujko, and A. Zolciak-Siwinska, *Risk of isolated nodal failure for non-small cell lung cancer (NSCLC) treated with the elective nodal irradiation (ENI) using 3D-conformal radiotherapy (3D-CRT) techniques - A retrospective analysis.* Acta Oncologica, 2008. 47: p. 95-103.
252. Crvenkova, S. and V. Krstevska, *Sequential chemoradiotherapy compared with concurrent chemoradiotherapy in locally advanced non-small cell lung cancer: our experience.* Makedonska Akademija na Naukite i Umetnostite Oddelenie Za Bioloshki i Meditsinski Nauki Prilozi, 2009. 30: p. 197-207.
253. De Jaeger, K., et al., *Significance of plasma transforming growth factor-beta levels in radiotherapy for non-small-cell lung cancer.* International Journal of Radiation Oncology Biology Physics, 2004. 58: p. 1378-1387.
254. Haasbeek, C.J.A., et al., *Stage I nonsmall cell lung cancer in patients aged >=75 years: Outcomes after stereotactic radiotherapy.* Cancer, 2010. 116: p. 406-414.
255. Qasim, M., *Systemic radiation and split-course radiotherapy for non-small-cell bronchial carcinoma.* Clinical Radiology, 1986. 37: p. 51-53.
256. Yuan, S.T., et al., *Timing and intensity of changes in FDG uptake with symptomatic esophagitis during radiotherapy or chemo-radiotherapy.* Radiation Oncology, 2014. 9 (1) (no pagination).
257. Newlin, H.E., et al., *Unresectable Squamous Cell Carcinoma of the Lung: An Outcomes Study.* International Journal of Radiation Oncology Biology Physics, 2009. 74: p. 370-376.
258. Valdes, G., et al., *Using machine learning to predict radiation pneumonitis in patients with stage I non-small cell lung cancer treated with stereotactic body radiation therapy.* Phys Med Biol, 2016. 61: p. 6105-20.
259. Morth, C., et al., *Validation and optimization of a predictive model for radiation pneumonitis in patients with lung cancer.* Oncol Lett, 2016. 12: p. 1144-1148.
