# Supplementary material for: Predicting Outcomes From Radical Radiotherapy for Non-small Cell Lung Cancer: A Systematic Review of the Existing Literature
Source: Front Oncol. 2018 Oct 10;8:433. doi: 10.3389/fonc.2018.00433 (PMC6191477; doi:10.3389/fonc.2018.00433)
Supplement: Supplementary file 2 [file Data_Sheet_2.DOCX]

Appendix B

References of Excluded studies

- **Reason for record exclusion:** s*ample size <50;* N = 8 [1-8]

1. Walker, M.J., et al., *Discovery and Validation of Predictive Biomarkers of Survival for Non-small Cell Lung Cancer Patients Undergoing Radical Radiotherapy: Two Proteins With Predictive Value.* EBioMedicine, 2015. 2(8): p. 841-50.
2. van Elmpt, W., et al., *Response assessment using 18F-FDG PET early in the course of radiotherapy correlates with survival in advanced-stage non-small cell lung cancer.* Journal of Nuclear Medicine, 2012. 53(10): p. 1514-20.
3. Hamamoto, Y., et al., *Relationship between pretreatment FDG uptake and local control after stereotactic body radiotherapy in stage I non-small-cell lung cancer: the preliminary results.* Japanese Journal of Clinical Oncology, 2011. 41(4): p. 543-7.
4. Chen, M., et al., *Long-term results of high-dose conformal radiotherapy for patients with medically inoperable T1-3N0 non-small-cell lung cancer: is low incidence of regional failure due to incidental nodal irradiation?* International Journal of Radiation Oncology, Biology, Physics, 2006. 64(1): p. 120-6.
5. Oshita, F., et al., *A feasibility study of continuous etoposide infusion combined with thoracic radiation for non-small cell lung cancer.* Oncology Reports, 1999. 6(2): p. 263-8.
6. Liu, M., et al., *Individual isotoxic radiation dose escalation based on V20 and advanced technologies benefits unresectable stage III non-small cell lung cancer patients treated with concurrent chemoradiotherapy: long term follow-up.* Oncotarget, 2017.
7. Schmitt, J.D., G.W. Warren, and I.Z. Wang, *Potential increase in biological effectiveness from field timing optimization for stereotactic body radiation therapy.* Medical Physics, 2012. 39: p. 2956-63.
8. Vos, C.G., et al., *Tumor size does not predict pathological complete response rates after pre-operative chemoradiotherapy for non-small cell lung cancer.* Acta Oncologica, 2013. 52: p. 676-678.

- **Reason for record exclusion:** population was not predominantly (>95% of population) stage IA, IB, IIA, IIB, IIIA, and/or IIIB NSCLC or an analyzed subgroup of these stages; N = 72 [9-80]

1. Wang, R., et al., *MicroRNA-148b is a potential prognostic biomarker and predictor of response to radiotherapy in non-small-cell lung cancer.* Journal of Physiology & Biochemistry, 2016. 72(2): p. 337-43.
2. Tolia, M., et al., *Prognostic Significance of Serum Inflammatory Response Markers in Newly Diagnosed Non-Small Cell Lung Cancer before Chemoirradiation.* BioMed Research International, 2015. 2015: p. 485732.
3. Kim, S.H., et al., *Clinical Significance of ABCG2 Haplotype-tagging Single Nucleotide Polymorphisms in Patients With Unresectable Non-Small Cell Lung Cancer Treated With First-line Platinum-based Chemotherapy.* American Journal of Clinical Oncology, 2015. 38(3): p. 294-9.
4. Holgersson, G., et al., *Effect of Increased Radiotoxicity on Survival of Patients with Non-small Cell Lung Cancer Treated with Curatively Intended Radiotherapy.* Anticancer Research, 2015. 35(10): p. 5491-7.
5. Gdowicz-Klosok, A., et al., *The SIPA1 -313A>G polymorphism is associated with prognosis in inoperable non-small cell lung cancer.* Tumour Biology, 2015. 36(2): p. 1273-8.
6. Farr, K.P., et al., *Inclusion of functional information from perfusion SPECT improves predictive value of dose-volume parameters in lung toxicity outcome after radiotherapy for non-small cell lung cancer: A prospective study.* Radiotherapy & Oncology, 2015. 117(1): p. 9-16.
7. Wen, J., et al., *Genetic variants of the LIN28B gene predict severe radiation pneumonitis in patients with non-small cell lung cancer treated with definitive radiation therapy.* European Journal of Cancer, 2014. 50(10): p. 1706-16.
8. van Oorschot, B., et al., *Survival and prognostic factors after moderately hypofractionated palliative thoracic radiotherapy for non-small cell lung cancer.* Strahlentherapie und Onkologie, 2014. 190(3): p. 270-5.
9. Tucker, S.L., et al., *Is there an impact of heart exposure on the incidence of radiation pneumonitis? Analysis of data from a large clinical cohort.* Acta Oncologica, 2014. 53(5): p. 590-6.
10. Castillo, R., et al., *Pre-radiotherapy FDG PET predicts radiation pneumonitis in lung cancer.* Radiation Oncology, 2014. 9: p. 74.
11. Xiong, H., et al., *ATM polymorphisms predict severe radiation pneumonitis in patients with non-small cell lung cancer treated with definitive radiation therapy.* International Journal of Radiation Oncology, Biology, Physics, 2013. 85(4): p. 1066-73.
12. Sharieff, W., et al., *Predicting 2-year survival for radiation regimens in advanced non-small cell lung cancer.* Clinical Oncology (Royal College of Radiologists), 2013. 25(12): p. 697-705.
13. Pang, Q., et al., *Functional promoter variant rs2868371 of HSPB1 is associated with risk of radiation pneumonitis after chemoradiation for non-small cell lung cancer.* International Journal of Radiation Oncology, Biology, Physics, 2013. 85(5): p. 1332-9.
14. Eblan, M.J., et al., *Brachial plexopathy in apical non-small cell lung cancer treated with definitive radiation: dosimetric analysis and clinical implications.* International Journal of Radiation Oncology, Biology, Physics, 2013. 85(1): p. 175-81.
15. Xu, T., et al., *HSPB1 gene polymorphisms predict risk of mortality for US patients after radio(chemo)therapy for non-small cell lung cancer.* International Journal of Radiation Oncology, Biology, Physics, 2012. 84(2): p. e229-35.
16. Niu, X., et al., *A study of ethnic differences in TGFbeta1 gene polymorphisms and effects on the risk of radiation pneumonitis in non-small-cell lung cancer.* Journal of Thoracic Oncology: Official Publication of the International Association for the Study of Lung Cancer, 2012. 7(11): p. 1668-75.
17. Holgersson, G., et al., *Swedish Lung Cancer Radiation Study Group: predictive value of age at diagnosis for radiotherapy response in patients with non-small cell lung cancer.* Acta Oncologica, 2012. 51(6): p. 759-67.
18. Gomez, D.R., et al., *Predictors of high-grade esophagitis after definitive three-dimensional conformal therapy, intensity-modulated radiation therapy, or proton beam therapy for non-small cell lung cancer.* International Journal of Radiation Oncology, Biology, Physics, 2012. 84(4): p. 1010-6.
19. Yin, M., et al., *Polymorphisms of homologous recombination genes and clinical outcomes of non-small cell lung cancer patients treated with definitive radiotherapy.* PLoS ONE [Electronic Resource], 2011. 6(5): p. e20055.
20. Petit, S.F., et al., *[18F]fluorodeoxyglucose uptake patterns in lung before radiotherapy identify areas more susceptible to radiation-induced lung toxicity in non-small-cell lung cancer patients.* International Journal of Radiation Oncology, Biology, Physics, 2011. 81(3): p. 698-705.
21. Lopez Guerra, J.L., et al., *Functional promoter rs2868371 variant of HSPB1 associates with radiation-induced esophageal toxicity in patients with non-small-cell lung cancer treated with radio(chemo)therapy.* Radiotherapy & Oncology, 2011. 101(2): p. 271-7.
22. Holgersson, G., et al., *Swedish lung cancer radiation study group: predictive value of histology for radiotherapy response in patients with non-small cell lung cancer.* European Journal of Cancer, 2011. 47(16): p. 2415-21.
23. Guan, X., et al., *TNFRSF1B +676 T>G polymorphism predicts survival of non-small cell lung cancer patients treated with chemoradiotherapy.* BMC Cancer, 2011. 11: p. 447.
24. Zhu, J., et al., *Analysis of acute radiation-induced esophagitis in non-small-cell lung cancer patients using the Lyman NTCP model.* Radiotherapy & Oncology, 2010. 97(3): p. 449-54.
25. Bradley, J.D., et al., *Stereotactic body radiation therapy for early-stage non-small-cell lung cancer: the pattern of failure is distant.* International Journal of Radiation Oncology, Biology, Physics, 2010. 77(4): p. 1146-50.
26. Jin, H., et al., *Dose-volume thresholds and smoking status for the risk of treatment-related pneumonitis in inoperable non-small cell lung cancer treated with definitive radiotherapy.* Radiotherapy & Oncology, 2009. 91(3): p. 427-32.
27. Tucker, S.L., et al., *Analysis of radiation pneumonitis risk using a generalized Lyman model.[Erratum appears in Int J Radiat Oncol Biol Phys. 2010 Sep 1;78(1):316-7 Note: Dosage error in article text].* International Journal of Radiation Oncology, Biology, Physics, 2008. 72(2): p. 568-74.
28. Gayou, O., et al., *A genetic algorithm for variable selection in logistic regression analysis of radiotherapy treatment outcomes.* Medical Physics, 2008. 35(12): p. 5426-33.
29. Wang, S., et al., *Analysis of clinical and dosimetric factors associated with treatment-related pneumonitis (TRP) in patients with non-small-cell lung cancer (NSCLC) treated with concurrent chemotherapy and three-dimensional conformal radiotherapy (3D-CRT).[Erratum appears in Int J Radiat Oncol Biol Phys. 2007 Apr 1;67(5):1606].* International Journal of Radiation Oncology, Biology, Physics, 2006. 66(5): p. 1399-407.
30. Giraud, P., et al., *Probability of mediastinal involvement in non-small-cell lung cancer: a statistical definition of the clinical target volume for 3-dimensional conformal radiotherapy?* International Journal of Radiation Oncology, Biology, Physics, 2006. 64(1): p. 127-35.
31. Sarihan, S., et al., *Evaluation of infections in non-small cell lung cancer patients treated with radiotherapy.* Cancer Detection & Prevention, 2005. 29(2): p. 181-8.
32. Brattstrom, D., et al., *Serum VEGF and bFGF adds prognostic information in patients with normal platelet counts when sampled before, during and after treatment for locally advanced non-small cell lung cancer.* Lung Cancer, 2004. 43(1): p. 55-62.
33. Willner, J., et al., *Dose, volume, and tumor control prediction in primary radiotherapy of non-small-cell lung cancer.* International Journal of Radiation Oncology, Biology, Physics, 2002. 52(2): p. 382-9.
34. Celebioglu, B., et al., *High dose rate endobronchial brachytherapy effectively palliates symptoms due to inoperable lung cancer.* Japanese Journal of Clinical Oncology, 2002. 32(11): p. 443-8.
35. Antonadou, D., *Radiotherapy or chemotherapy followed by radiotherapy with or without amifostine in locally advanced lung cancer.* Seminars in Radiation Oncology, 2002. 12(1 Suppl 1): p. 50-8.
36. Bergqvist, M., et al., *P53 auto-antibodies in non-small cell lung cancer patients can predict increased life expectancy after radiotherapy.* Anticancer Research, 1998. 18(3B): p. 1999-2002.
37. Paesmans, M., et al., *Response to chemotherapy has predictive value for further survival of patients with advanced non-small cell lung cancer: 10 years experience of the European Lung Cancer Working Party.* European Journal of Cancer, 1997. 33(14): p. 2326-32.
38. Takigawa, N., et al., *Prognostic factors for patients with advanced non-small cell lung cancer: univariate and multivariate analyses including recursive partitioning and amalgamation.* Lung Cancer, 1996. 15(1): p. 67-77.
39. Komaki, R., et al., *Analysis of early and late deaths on RTOG non-small cell carcinoma of the lung trials: comparison with CALGB 8433.* Lung Cancer, 1993. 10(3-4): p. 189-97.
40. Koh, W.J., et al., *Neutron vs. photon radiation therapy for inoperable regional non-small cell lung cancer: results of a multicenter randomized trial.* International Journal of Radiation Oncology, Biology, Physics, 1993. 27(3): p. 499-505.
41. Graham, M.V., et al., *Comparison of prognostic factors and survival among black patients and white patients treated with irradiation for non-small-cell lung cancer.* Journal of the National Cancer Institute, 1992. 84(22): p. 1731-5.
42. Chatani, M., et al., *Radiation therapy combined with or without chemotherapy for limited extent of non-small cell lung carcinoma.* Strahlentherapie und Onkologie, 1988. 164(6): p. 330-6.
43. Eagan, R.T., et al., *Thoracic radiation therapy and Adriamycin/cisplatin-containing chemotherapy for locally advanced non-small-cell lung cancer.* Cancer Clinical Trials, 1981. 4(4): p. 381-8.
44. Yu, Y., et al., *Advances in dosimetry and biological predictors of radiation-induced esophagitis.* OncoTargets and Therapy, 2016. 9: p. 597-603.
45. Han, S., et al., *Analysis of Clinical and Dosimetric Factors Influencing Radiation-Induced Lung Injury in Patients with Lung Cancer.* J Cancer, 2015. 6: p. 1172-8.
46. Arriagada, R., et al., *Astro plenary: Effect of chemotherapy on locally advanced non-small cell lung carcinoma: A randomized study of 353 patients.* International Journal of Radiation Oncology Biology Physics, 1991. 20: p. 1183-1190.
47. Christoph, D.C., et al., *Bv-tubulin expression is associated with outcome following taxane-based chemotherapy in non-small cell lung cancer.* British Journal of Cancer, 2012. 107: p. 823-830.
48. Petrovic, M., et al., *Chromogranin a tissue expression as a prognostic factor in advanced non small cell lung cancer.* Serbian Journal of Experimental and Clinical Research, 2008. 9: p. 137-142.
49. Zhang, Z.C., et al., *Clinical and dosimetric risk factors of acute esophagitis in patients treated with 3-dimensional conformal radiotherapy for non-small-cell lung cancer.* American Journal of Clinical Oncology: Cancer Clinical Trials, 2010. 33: p. 271-275.
50. Takeda, A., et al., *Comparison of clinical, tumour-relatedand dosimetric factors in grade 0-1, grade2 and grade 3 radiation pneumonitis after stereotactic body radiotherapy for lung tumours.* British Journal of Radiology, 2012. 85: p. 636-642.
51. Nieder, C., et al., *Development of a score predicting survival after palliative reirradiation.* Journal of Oncology, 2014. 2014 (no pagination).
52. Jochems, A., et al., *Distributed learning: Developing a predictive model based on data from multiple hospitals without data leaving the hospital - A real life proof of concept.* Radiotherapy and Oncology, 2016. 121: p. 459-467.
53. Wang, S.W., et al., *Effect of image-guided hypofractionated stereotactic radiotherapy on peripheral non-small-cell lung cancer.* Onco Targets Ther, 2016. 9: p. 4993-5003.
54. DeGrendele, H., et al., *Fluorodeoxyglucose positron emission tomography as a staging and prognostic tool in non-small-cell lung cancer.* Clinical Lung Cancer, 2003. 4: p. 213-216.
55. Liu, B., et al., *Genetic variants in the plasminogen activator inhibitor-1 gene are associated with an increased risk of radiation pneumonitis in lung cancer patients.* Cancer Medicine, 2017. 6: p. 681-688.
56. Jingu, K., et al., *Influence of etoposide on the local effect to radiation in non-small cell lung cancer. A randomized prospective study.* Journal of JASTRO, 1993. 5: p. 141-149.
57. Kilburn, J.M., et al., *Is a Clinical Target Volume (CTV) Necessary in the Treatment of Lung Cancer in the Modern Era Combining 4-D Imaging and Image-guided Radiotherapy (IGRT)?* Cureus, 2016. 8: p. e466.
58. Fang, P., et al., *Outcomes and toxicity following high-dose radiation therapy in 15 fractions for non-small cell lung cancer.* Pract Radiat Oncol, 2017.
59. Huber, R.M., et al., *Palliative endobronchial brachytherapy for central lung tumors: A prospective, randomized comparison of two fractionation schedules.* Chest, 1995. 107: p. 463-470.
60. Schroder, C., et al., *Patient's quality of life after high-dose radiation therapy for thoracic carcinomas: Changes over time and influence on clinical outcome Lebensqualitat von Patienten nach Hochdosis-Strahlentherapie thorakaler Karzinome: Zeitlicher Verlauf und Einfluss auf das klinische Ergebnis.* Strahlentherapie und Onkologie, 2017. 193: p. 132-140.
61. Rechnitzer, S., et al., *Pretreatment prognostic factors for survival in non-small-cell lung cancer: A multivariate analysis of 229 patients.* Onkologie, 1998. 21: p. 204-210.
62. Ohlhauser, C., et al., *Prognostic factors for survival in inoperable non-small-cell lung cancer: A multivariate regression analysis of 456 patients with radiation therapy.* Onkologie, 1997. 20: p. 126-131.
63. Kanzaki, R., et al., *PSF1 (Partner of SLD Five 1) is a Prognostic Biomarker in Patients with Non-small Cell Lung Cancer Treated with Surgery Following Preoperative Chemotherapy or Chemoradiotherapy.* Annals of Surgical Oncology, 2016. 23: p. 4093-4100.
64. Minet, P., P. Bartsch, and P. Chevalier, *Quality of life of inoperable non-small cell lung carcinoma. A randomized phase II clinical study comparing radiotherapy alone and combined radio-chemotherapy.* Radiotherapy and Oncology, 1987. 8: p. 217-230.
65. Ranson, M., et al., *Randomized trial of paclitaxel plus supportive care versus supportive care for patients with advanced non-small-cell lung cancer.* Journal of the National Cancer Institute, 2000. 92: p. 1074-1080.
66. Sau, S., et al., *Retrospective analysis of the clinical and demographic variables on the outcomes after second-line treatment in advanced non-small cell lung cancer.* Indian J Med Paediatr Oncol, 2014. 34: p. 274-9.
67. Zhang, Z., et al., *Risk factors of radiation-induced acute esophagitis in non-small cell lung cancer patients treated with concomitant chemoradiotherapy.* Radiation Oncology, 2014. 9 (1) (no pagination).
68. Xiao, Y., et al., *Single-nucleotide polymorphisms of TGFbeta1 and ATM associated with radiation-induced pneumonitis: a prospective cohort study of thoracic cancer patients in China.* Int J Clin Exp Med, 2015. 8: p. 16403-13.
69. Nieder, C., et al., *Survival after palliative radiotherapy in geriatric cancer patients.* Anticancer Research, 2014. 34: p. 6641-6645.
70. Kharofa, J. and E. Gore, *Symptomatic radiation pneumonitis in elderly patients receiving thoracic irradiation.* Clinical Lung Cancer, 2013. 14: p. 283-287.
71. Middelburg, J.G., et al., *Timed Get Up and Go Test and Geriatric 8 Scores and the Association With (Chemo-)Radiation Therapy Noncompliance and Acute Toxicity in Elderly Cancer Patients.* Int J Radiat Oncol Biol Phys, 2017. 98: p. 843-849.
72. Brocato, N., et al., *Treatment of Non-Small cell lung cancer with ifosfamide (IFO) + 4'-Epiadriamycin (EPI) + platinum vs. IFO + EPI: A GETLAC study.* Oncology, 1995. 52: p. 24-31.

- **Reason for record exclusion:** t*reatment was not RRT -- patients had surgery before radiotherapy;* N = 36 [81-116]

1. Wang, T.L., et al., *Dual Specificity Phosphatase 6 (DUSP6) Polymorphism Predicts Prognosis of Inoperable Non-Small Cell Lung Cancer after Chemoradiotherapy.* Clinical Laboratory, 2016. 62(3): p. 301-10.
2. Chang, J.Y., et al., *Stereotactic ablative radiation therapy for centrally located early stage or isolated parenchymal recurrences of non-small cell lung cancer: how to fly in a "no fly zone".* International Journal of Radiation Oncology, Biology, Physics, 2014. 88(5): p. 1120-8.
3. Uyterlinde, W., et al., *Prediction of acute toxicity grade > 3 in patients with locally advanced non-small-cell lung cancer receiving intensity modulated radiotherapy and concurrent low-dose Cisplatin.* Clinical Lung Cancer, 2013. 14(5): p. 541-8.
4. Maraz, A., et al., *Acute oesophageal toxicity related to paclitaxel-based concurrent chemoradiotherapy for non-small cell lung cancer.* Anticancer Research, 2013. 33(4): p. 1737-41.
5. Farr, K.P., et al., *Development of radiation pneumopathy and generalised radiological changes after radiotherapy are independent negative prognostic factors for survival in non-small cell lung cancer patients.* Radiotherapy & Oncology, 2013. 107(3): p. 382-8.
6. Zhang, X., et al., *Positron emission tomography for assessing local failure after stereotactic body radiotherapy for non-small-cell lung cancer.* International Journal of Radiation Oncology, Biology, Physics, 2012. 83(5): p. 1558-65.
7. Rades, D., et al., *The prognostic impact of tumor cell expression of estrogen receptor-alpha, progesterone receptor, and androgen receptor in patients irradiated for nonsmall cell lung cancer.* Cancer, 2012. 118(1): p. 157-63.
8. Butkiewicz, D., et al., *Influence of DNA repair gene polymorphisms on prognosis in inoperable non-small cell lung cancer patients treated with radiotherapy and platinum-based chemotherapy.* International Journal of Cancer, 2012. 131(7): p. E1100-8.
9. Phernambucq, E.C., et al., *Outcomes of concurrent chemoradiotherapy in patients with stage III non-small-cell lung cancer and significant comorbidity.* Annals of Oncology, 2011. 22(1): p. 132-8.
10. Chen, A.B., et al., *Survival outcomes after radiation therapy for stage III non-small-cell lung cancer after adoption of computed tomography-based simulation.* Journal of Clinical Oncology, 2011. 29(17): p. 2305-11.
11. Aumont-le Guilcher, M., et al., *High-dose-rate brachytherapy for non-small-cell lung carcinoma: a retrospective study of 226 patients.* International Journal of Radiation Oncology, Biology, Physics, 2011. 79(4): p. 1112-6.
12. McGovern, S.L., et al., *Is sex associated with the outcome of patients treated with radiation for nonsmall cell lung cancer?* Cancer, 2009. 115(14): p. 3233-42.
13. Rube, C.E., et al., *Cytokine plasma levels: reliable predictors for radiation pneumonitis?* PLoS ONE [Electronic Resource], 2008. 3(8): p. e2898.
14. Rades, D., et al., *Effect of smoking during radiotherapy, respiratory insufficiency, and hemoglobin levels on outcome in patients irradiated for non-small-cell lung cancer.* International Journal of Radiation Oncology, Biology, Physics, 2008. 71(4): p. 1134-42.
15. Fuwa, N., et al., *Identifying patients with peripheral-type early non-small cell lung cancer (T1N0M0) for whom irradiation of the primary focus alone could lead to successful treatment.* British Journal of Radiology, 2008. 81(970): p. 815-20.
16. Yoon, S.M., et al., *The polymorphism and haplotypes of XRCC1 and survival of non-small-cell lung cancer after radiotherapy.* International Journal of Radiation Oncology, Biology, Physics, 2005. 63(3): p. 885-91.
17. Kong, F.M., et al., *High-dose radiation improved local tumor control and overall survival in patients with inoperable/unresectable non-small-cell lung cancer: long-term results of a radiation dose escalation study.* International Journal of Radiation Oncology, Biology, Physics, 2005. 63(2): p. 324-33.
18. Ahn, S.J., et al., *Dosimetric and clinical predictors for radiation-induced esophageal injury.* International Journal of Radiation Oncology, Biology, Physics, 2005. 61(2): p. 335-47.
19. Maurel, J., et al., *Prognostic impact of bulky mediastinal lymph nodes (N2>2.5 cm) in patients with locally advanced non-small-cell lung cancer (LA-NSCLC) treated with platinum-based induction chemotherapy.* Lung Cancer, 2000. 30(2): p. 107-16.
20. Komaki, R., et al., *Outcome predictors for 143 patients with superior sulcus tumors treated by multidisciplinary approach at the University of Texas M. D. Anderson Cancer Center.* International Journal of Radiation Oncology, Biology, Physics, 2000. 48(2): p. 347-54.
21. Lutz, S.T., et al., *A retrospective quality of life analysis using the Lung Cancer Symptom Scale in patients treated with palliative radiotherapy for advanced nonsmall cell lung cancer.* International Journal of Radiation Oncology, Biology, Physics, 1997. 37(1): p. 117-22.
22. Lotayef, M., et al., *A clinico-epidemilogical study of cases of locally advanced non-small cell lung cancer (NSCLC) that received radiotherapy at NCI Cairo in the period from 2001-2010.* Journal of Cancer Science and Therapy, 2014. 6: p. 151-155.
23. Nakamichi, S., et al., *Comparison of Radiotherapy and Chemoradiotherapy for Locoregional Recurrence of Non-small-cell Lung Cancer Developing After Surgery.* Clin Lung Cancer, 2017.
24. Luciani, A., et al., *Dose intensity correlate with survival in elderly patients treated with chemotherapy for advanced non-small cell lung cancer.* Lung Cancer, 2009. 66: p. 94-96.
25. Dubey, S., et al., *EGFR dinucleotide repeat polymorphism as a prognostic indicator in non-small cell lung cancer.* Journal of Thoracic Oncology, 2006. 1: p. 406-412.
26. Robnett, T.J., et al., *Factors affecting the risk of brain metastases after definitive chemoradiation for locally advanced non-small-cell lung carcinoma.* Journal of Clinical Oncology, 2001. 19: p. 1344-1349.
27. Eschmann, S.M., et al., *Impact of staging with 18F-FDG-PET on outcome of patients with stage III non-small cell lung cancer: PET identifies potential survivors.* European Journal of Nuclear Medicine and Molecular Imaging, 2007. 34: p. 54-59.
28. Akthar, A.S., et al., *Limitations of PET/CT in the Detection of Occult N1 Metastasis in Clinical Stage I(T1-2aN0) Non-Small Cell Lung Cancer for Staging Prior to Stereotactic Body Radiotherapy.* Technol Cancer Res Treat, 2016. 16: p. 15-21.
29. Li, F., et al., *A meta-analysis identifies ERCC1 gene polymorphism as a predictor of better patient response to treatment with radiochemotherapy.* Cancer Chemotherapy and Pharmacology, 2016. 77: p. 1183-1191.
30. Crino, L. and F. Cappuzzo, *Multimodality therapy for non-small cell lung cancer (NSCLC): Ongoing Italian experiences in the adjuvant and neoadjuvant settings.* Lung Cancer, 2001. 34: p. 45-47.
31. Paripati, H.R., et al., *Multimodality therapy improves survival in elderly patients with locally advanced non-small cell lung cancer-A retrospective analysis.* Journal of Geriatric Oncology.
32. Kim, H.K., et al., *Outcomes of neoadjuvant concurrent chemoradiotherapy followed by surgery for non-small-cell lung cancer with N2 disease.* Lung Cancer, 2016. 96: p. 56-62.
33. Verma, V., et al., *Outcomes of Stereotactic Body Radiotherapy for T1-T2N0 Small Cell Carcinoma According to Addition of Chemotherapy and Prophylactic Cranial Irradiation: A Multicenter Analysis.* Clin Lung Cancer, 2017.
34. Emami, B., et al., *Radiation therapy for intrathoracic recurrence of non-small cell lung cancer.* American Journal of Clinical Oncology: Cancer Clinical Trials, 1997. 20: p. 46-50.
35. Bott, M.J., et al., *Role for surgical resection in the multidisciplinary treatment of stage IIIB non-small cell lung cancer.* Annals of Thoracic Surgery, 2015. 99: p. 1921-1928.
36. Hanson, S., et al., *Treatment outcomes in stage IIIA non-small-cell lung cancer in a community cancer center.* Journal of Community and Supportive Oncology, 2015. 13: p. 292-295.

- **Reason for record exclusion:** did not investigate risk factor(s) and RRT association; N = 5 [117-121, 71]

1. Chowdhry, V.K., et al., Complications from computed tomography-guided core needle biopsy for patients receiving stereotactic body radiation therapy for early-stage lesions of the lung. Clinical Lung Cancer, 2014. 15(4): p. 302-6.
2. Ball, D.L., et al., Stage is not a reliable indicator of tumor volume in non-small cell lung cancer: a preliminary analysis of the Trans-Tasman Radiation Oncology Group 99-05 database. Journal of Thoracic Oncology: Official Publication of the International Association for the Study of Lung Cancer, 2006. 1(7): p. 667-72.
3. Steuer, C.E., et al., *Comparison of Concurrent Use of Thoracic Radiation With Either Carboplatin-Paclitaxel or Cisplatin-Etoposide for Patients With Stage III Non-Small-Cell Lung Cancer: A Systematic Review.* JAMA Oncol, 2016.
4. El-Hadaad, H.A., H.A. Wahba, and E. Toson, *Induction chemotherapy followed by weekly paclitaxel and carboplatin with concurrent radiotherapy in inoperable stage III non-small cell lung cancers: Results of a phase II trial.* Chinese-German Journal of Clinical Oncology, 2013. 12: p. P56-P60.
5. Xu, Y., et al., *Simultaneous integrated boost intensity-modulated radiotherapy for locally advanced non-small cell lung cancer in Chinese population: A retrospective study.* Oncotarget, 2017.

- **Reason for record exclusion:** did not clinical impact (effectiveness and harms) of response to RRT risk prediction guided patient management; N = 1 [122]

1. Vulto, A.J., et al., The influence of age and comorbidity on receiving radiotherapy as part of primary treatment for cancer in South Netherlands, 1995 to 2002.[Erratum appears in Cancer. 2006 Dec 15;107(12):2960]. Cancer, 2006. 106(12): p. 2734-42.

- **Reason for record exclusion:** language other than English; N = 45 [123-167]

1. Li, F., et al., *[Role of EGFR mutation status in patients with stage III non-squamous non-small cell lung cancer treated with chemoradiotherapy].* Chinese Journal of Lung Cancer, 2011. 14(9): p. 715-8.
2. Xing, J., et al., *[Relationship of dose-volume histogram parameters and computed tomography grading of radiation-induced lung injury in patients with non-small cell lung cancer treated by three-dimensional conformal radiotherapy].* Chung-Hua Chung Liu Tsa Chih [Chinese Journal of Oncology], 2008. 30(9): p. 676-81.
3. Zhu, X.Z., et al., *[Prognosis of locally advanced non small cell lung cancer treated with three dimentional conformal radiotherapy].* Chung-Hua Chung Liu Tsa Chih [Chinese Journal of Oncology], 2007. 29(10): p. 748-53.
4. Chen, T.F., et al., *[Detection and prognostic significance of micrometastasis in peripheral blood of patients with non-small cell lung cancer treated by chemo-radiation therapy].* Chung-Hua Chung Liu Tsa Chih [Chinese Journal of Oncology], 2007. 29(5): p. 365-8.
5. Jaen Olasolo, J., et al., *[Non-small cell lung cancer. Survival after radiotherapy and prognostic factors].* Archivos de Bronconeumologia, 2003. 39(2): p. 81-6.
6. Xiao, A., et al., *[Radiotherapy for 308 patients with non-small cell lung cancer (NSCLC)].* Chinese Journal of Lung Cancer, 2001. 4(2): p. 134-6.
7. Hu, Y., A. Zhong, and X. Yang, *[Prognostic value of serum CA125 level in patients with advanced non-small cell lung cancer].* Chung-Hua Chung Liu Tsa Chih [Chinese Journal of Oncology], 2000. 22(1): p. 30-1.
8. Kodaira, T., et al., *[Clinical efficacy of concurrent carboplatin, etoposide, and definitive radiotherapy for stage III non-small-cell lung cancer: consideration as to therapeutic outcomes and morbidity].* Nippon Igaku Hoshasen Gakkai Zasshi - Nippon Acta Radiologica, 1998. 58(9): p. 497-502.
9. Wurschmidt, F., et al., *[The radiotherapy of inoperable non-small-cell bronchial carcinoma. A retrospective analysis of 427 cases].* Strahlentherapie und Onkologie, 1994. 170(5): p. 302-4.
10. Fuwa, N., et al., *[An analysis of the prognostic factors of stage I non-small cell lung cancer treated with radiation therapy].* Nippon Gan Chiryo Gakkai Shi - Journal of Japan Society for Cancer Therapy, 1987. 22(9): p. 2218-24.
11. Yin, L.J., et al., *Association of survivin and K-ras protein with radiosensitivity in non-small cell lung cancer tissues.* Chinese Journal of Cancer Prevention and Treatment, 2010. 17: p. 1080-1082.
12. Zhang, L., *Chemotherapy on nonresectable stage III N2 non-small cell lung cancer.* Chinese Journal of Lung Cancer, 2008. 11: p. 657-662.
13. Zhan, Y., C. Huang, and J. Huang, *Clinical analysis of the effect of concurrent radiochemotherapy on 159 unresectable cases of stage III non-small cell lung cancer.* Chinese Journal of Clinical Oncology, 2009. 36: p. 1274-1276.
14. Li, G.Z., et al., *The clinical comparative study on hydroxycomptothecin combined with cocurrent hyperfractionated radiotherapy for unresectable stage III non-small cell lung cancer.* Tumor, 2008. 28: p. 156-158.
15. Yang, W., H. Wang, and L. Tang, *Clinical factor analysis of radiotherapy effect for 318 patients with non-small cell lung cancer.* Chinese Journal of Clinical Oncology, 2005. 32: p. 40-42.
16. Tan, X., et al., *Clinical observation on the effects of weekly docetaxel combined with three-dimensional conformal radiotherapy in the treatment of elderly patients with non-small cell lung cancer.* Anti-Tumor Pharmacy, 2013. 3: p. 451-454.
17. Morita, K. and N. Fuwa, *Clinical significance of tumor size as a prognostic factor in radiotherapy for non-small cell lung cancer.* Japanese Journal of Lung Cancer, 1990. 30: p. 231-238.
18. Wu, H., et al., *Clinical trial of concurrent low-dose chemotherapy plus radiation vs sequential chemoradiotherapy for unresectable stage III non-small cell lung cancer.* Chinese Journal of Lung Cancer, 2006. 9: p. 283-285.
19. Zhang, Z., et al., *Concurrent chemotherapy and intensity-modulated radiation therapy positioned by PET/CT for patients with locally advanced non-small cell lung cancer.* Chinese Journal of Clinical Oncology, 2013. 40: p. 930-933.
20. Ma, J., C. Han, and H. Zou, *Concurrent or sequential gemcitabine combined with radiotherapy for locally advanced non-small cell lung cancer.* Chinese Journal of Clinical Oncology, 2011. 38: p. 328-331.
21. Zhao, J., et al., *Correlation between the serum amyloid A level and overall survival and radiation pneumonitis in non-small cell lung cancer patients receiving thoracic radiotherapy.* Chinese Journal of Cancer Prevention and Treatment, 2016. 23: p. 1628-1633.
22. Varol, Y., et al., *The effect of consolidation chemotherapy for LA-NSCLC patients receiving concurrent chemoradiotherapy Es zamanli kemoradyoterapi alan lokal Ileri KHDAK hastalarda konsolidasyon kemoterapisinin etkinligi.* Journal of Clinical and Analytical Medicine, 2017. 8: p. 142-146.
23. Hu, W., et al., *Effect of protein tyrosine phosphatase non-receptor type 12 (PTPN12) downregulation on the radiosensitivity of non-small cell lung cancer.* Chinese Journal of Clinical Oncology, 2016. 43: p. 285-290.
24. Lan, Y., et al., *Efficacy of IMRT combined with docetaxel concurrent chemotherapy for elderly patients with local advanced non-small cell lung cancer.* Anti-Tumor Pharmacy, 2014. 4: p. 379-382.
25. Giraud, P., et al., *Estimation of the mediastinal involvement probability in non-small cell lung cancer: A statistical definition of the clinical target volume for 3-dimensional conformal radiotherapy? Estimation de la probabilite d'envahissement tumoral mediastinal: Une definition statistique du volume-cible anatomoclinique pour la radiotherapie conformationnelle des cancers bronchiques non a petites cellules?* Cancer/Radiotherapie, 2001. 5: p. 725-736.
26. Li, G., et al., *Expression and biological significance of Ku70 and Ku80 in non-small cell lung cancer tissues.* Chinese Journal of Cancer Prevention and Treatment, 2012. 19: p. 1628-1631.
27. Zhou, H., et al., *Impact of diabetes mellitus on clinicopathological factors and relation with radiation pneumonitis in 332 patients with lung cancer.* Zhong Nan da Xue Xue Bao. Yi Xue Ban = Journal of Central South University. Medical Sciences, 2013. 38: p. 138-41.
28. Shen, W., et al., *Impact of the gross tumor volume and radiotherapeutic dose on the survival of patients with locally advanced non-small cell lung cancer.* Chinese Journal of Clinical Oncology, 2012. 39: p. 278-282.
29. Zhang, C.W., N.K. Zhou, and B.B. Peng, *Influence of preoperative concurrent radiochemotherapy on resection rate and long-term of stage III non-small cell lung cancer.* Chinese Journal of Cancer Prevention and Treatment, 2010. 17: p. 221-223.
30. Xing, X.B., et al., *Intensity modulated radiation therapy combined with docetaxl chemotherapy for locally late stage non-small cell lung cancer.* Chinese Journal of Cancer Prevention and Treatment, 2011. 18: p. 1280-1282.
31. Xing, L.Q., et al., *Late-course three-dimensional conformal concurrent chemoradiotherapy for 31 patients with non-small cell lung carcinoma.* Tumor, 2008. 28: p. 1004-1007.
32. Qiao, W. and Y. Zhao, *The predictors of radiation-induced esophageal toxicity in patients with non-small cell lung cancer treated with three-dimensional conformal radiotherapy.* Chinese Journal of Clinical Oncology, 2005. 32: p. 1421-1423.
33. Kodama, A., et al., *Prognostic factors and long-term survival of non-resected cases with non-small cell lung cancer.* Japanese Journal of Lung Cancer, 1996. 36: p. 91-98.
34. Bao, P.T., et al., *Protective effect of ulinastatin on lung injury in patients with non-small cell lung cancer after radiation therapy.* Chinese Journal of New Drugs, 2014. 23: p. 2066-2070.
35. Wang, C., R. Wang, and W. Qiao, *A randomized prospective study on combined chemoradiotherapy for non-small cell lung cancer.* Zhongguo Fei Ai Za Zhi, 2000. 3: p. 330-2.
36. Lu, J., et al., *Randomized trial of hyperfractionated plus accelerated hyperfractionated radiation therapy with or without concomitant chemotherapy for stage III A / III B non-small-cell lung cancer: A preliminary result.* Chinese Journal of Lung Cancer, 2002. 5: p. 423-426.
37. Petrovic, M., I. Tomic, and D. Jovanovic, *Risk factors for brain metastases after definitive chemoradiation for locally advanced non-small cell lung cancer. [Serbian] Faktori rizika od pojave metastaza u mozgu kod lokalno odmaklog nemikrocelularnog karcinoma pluca nakon zavrsene hemio-zracne terapije.* Vojnosanitetski Pregled, 2009. 66: p. 876-880.
38. Durdux, C., et al., *The role of radiotherapy in inoperable non-small cell lung cancer: Study of a series of 381 patients Radiotherapie dans le traitement locoregional du cancer bronchique non a petites cellules localise inoperable: Resultats portant sur une serie de 381 patients.* Cancer/Radiotherapie, 1997. 1: p. 132-136.
39. Zhu, L. and R. Wu, *Sequential and concurrent radiochemotherapy for stage III non-small cell lung cancer.* Chinese Journal of Cancer Prevention and Treatment, 2009. 16: p. 1105-1107.
40. Wang, T.J., et al., *Short-term effects of Sipulin combined with radiotherapy on local advanced non-small cell lung cancer and its influence on immune function.* Tumor, 2007. 27: p. 493-495.
41. Osowiecka, K., et al., *Survival of patients treated with radiation for non-small-cell lung cancer in Samodzielny Publiczny Zaklad Opieki Zdrowotnej Ministerstwa Spraw Wewnetrznych z Warminsko-Mazurskim Centrum Onkologii in Olsztyn between 2003 and 2006 Przezycia chorych na niedrobnokomorkowego raka pluca leczonych napromienianiem w latach 2003-2006 w Samodzielnym Publicznym Zakladzie Opieki Zdrowotnej Ministerstwa Spraw Wewnetrznych z Warminsko-Mazurskim Centrum Onkologii w Olsztynie.* Nowotwory, 2015. 65: p. 14-22.
42. Wilhelm, R., et al., *Survival of primary irradiated patients with NSCLC in dependence of the pretherapeutical haemoglobin level Uberlebenszeiten ausschliesslich bestrahlter NSCLCL-Patienten: Die bedeutung des pratherapeutischen hamoglobinwerts.* Strahlentherapie und Onkologie, 1998. 174: p. 128-132.
43. Wang, X.C., et al., *Three-dimensional conformal radiotherapy combined with stereotactic radiotherapy for locally advanced non-small cell lung cancer: efficacy and complications.* Nan Fang Yi Ke Da Xue Xue Bao = Journal of Southern Medical University, 2008. 28: p. 1996-8.
44. Tsujino, K., *Ways to prevent severe and fatal radiation pneumonitis.* Japanese Journal of Clinical Radiology, 2015. 60: p. 1215-1219.
45. Villanueva, N., et al., *Weekly 70 Gy thoracic radiotheraphy concurrent with docetaxel or paclitaxel after induction chemotherapy of patients with locally advanced non-small cell lung cancer: Prospective study of tolerance and efficacy Radioterapia toracica con 70 Gy concurrente con docetaxel o paclitaxel semanal tras quimioterapia de induccion en pacientes con carcinoma de pulmon no microcitico localmente avanzado. Estudio prospectivo de tolerancia y eficacia.* Oncologia, 2006. 29: p. 412-418.

- **Reason for record exclusion:** reports is a non-systematic review, letter, editorial or case-report; N = 19 [168-186]

1. Bruner, D.W., et al., *Outcomes research in cancer clinical trial cooperative groups: the RTOG model.* Quality of Life Research, 2004. 13(6): p. 1025-41.
2. Choi, N., et al., *Predictive factors in radiotherapy for non-small cell lung cancer: present status.* Lung Cancer, 2001. 31(1): p. 43-56.
3. Armstrong, J. and C. McGibney, *The impact of three-dimensional radiation on the treatment of non-small cell lung cancer.* Radiotherapy & Oncology, 2000. 56(2): p. 157-67.
4. Chamberlain, M.C., *Are EGFR antagonists associated with increased central nervous system metastases in non-small-cell lung cancer?* Nature Clinical Practice Oncology, 2006. 3: p. 20-21.
5. Anonymous, *Association study between SNPs in the regulator of G-protein signaling genes and survival in non-small-cell lung cancer.* Pharmacogenomics, 2011. 12: p. 1517-1518.
6. Eberhardt, W.E.E., *Concurrent chemoradiotherapy in stage III non-small-cell lung cancer: What is the best regimen?* Journal of Clinical Oncology, 2015. 33: p. 532-533.
7. Kalbasi, A., G.L. Beatty, and A.T. Berman, *Expanding tumor lymphocytic infiltration as a prognostic tool to patients with NSCLC who are treated with radiotherapy?* Journal of Thoracic Oncology, 2016. 11: p. e141-e142.
8. Fuentes, R., et al., *Factors influencing survival in a prospective cohort of patients with non-small cell lung cancer: An updated assessment [4.* Clinical Oncology, 2004. 16: p. 583-584.
9. Rocco, G., *Hear! Hear! Against alLODDS, we might have a LiNeaR predictive model for prognosis of non-small cell lung cancer from pN+ nodal status.* Journal of Thoracic and Cardiovascular Surgery, 2017. 153: p. 710.
10. Casas, F. and B. Jeremic, *In regard to Bogart et al.: 70 Gy thoracic radiotherapy is feasible concurrent with chemotherapy for limited-stage non-small-cell lung cancer: Analysis of cancer and leukemia group B study 39808 (Int J Radiat Oncol Biol Phys 2004;59:460-468) [2.* International Journal of Radiation Oncology Biology Physics, 2004. 60: p. 1661-1662.
11. Shien, K., et al., *Is tumor location an independent prognostic factor in locally advanced non-small cell lung cancer treated with trimodality therapy?* Journal of Thoracic Disease, 2017. 9: p. E489-E491.
12. Jeremic, B., et al., *No role for trimodality therapy and consolidation chemotherapy compared with concurrent radiochemotherapy alone in stage III non-small-cell lung cancer.* Journal of Clinical Oncology, 2016. 34: p. 196-197.
13. Watine, J. and J.C. Charet, *Ohlhauser C, et al.: Prognostic factors for survival in inoperable non- small-cell lung cancer: A multivariate regression analysis of 456 patients with radiation therapy. Onkologie 1997;20:126-131 [1.* Onkologie, 1998. 21: p. 330-331.
14. Baka, S., et al., *Platinum-based chemotherapy with thoracic radiotherapy in stage III good performance status non-small cell lung cancer patients.* European Journal of Cancer, Supplement, 2005. 3: p. 41-50.
15. Holgersson, G., et al., *Radiosensitizing biological modifiers enhancing efficacy in non-small-cell lung cancer treated with radiotherapy.* Lung Cancer Management, 2013. 2: p. 251-255.
16. Zhao, J., et al., *Simple Factors Associated With Radiation-Induced Lung Toxicity After Stereotactic Body Radiation Therapy of the Thorax: A Pooled Analysis of 88 Studies.* International Journal of Radiation Oncology Biology Physics, 2016. 95: p. 1357-1366.
17. Daly, P.E. and D.L. Ball, *Tumor size and outcomes in non-small-cell lung carcinoma treated with radiotherapy: A critical review.* Lung Cancer Management, 2014. 3: p. 207-218.
18. Senthi, S., *Use of stereotactic body radiation therapy with salvage surgery to improve outcomes for early stage non-small cell lung cancer.* Journal of Thoracic and Cardiovascular Surgery, 2014. 148: p. 1760.
19. Dahele, M. and S. Senan, *What causes early mortality in patients with large tumors receiving radical chemo-radiotherapy for non-small cell lung cancer? in response to Ball et al.* Radiotherapy and Oncology, 2013. 109: p. 179-180.

- **Reason for record exclusion:** investigation is about average risk/incidence/odds of specific outcomes following RRT (i.e. fundamental prognosis research) but not about predictive factor risk assessment; N = 1 [187]

1. Brundage, M.D., et al., *Decision analysis in locally advanced non-small-cell lung cancer: is it useful?* Journal of Clinical Oncology, 1997. 15(3): p. 873-83.

- **Reason for record exclusion:** predicted outcome is not a pre-specified outcome; N = 77 [188-264]

1. Walraven, I., et al., *Long-term follow-up of patients with locally advanced non-small cell lung cancer receiving concurrent hypofractionated chemoradiotherapy with or without cetuximab.* Radiotherapy & Oncology, 2016. 118(3): p. 442-6.
2. Yu, Y., et al., *Role of gross tumor volume in the prognosis of non-small cell lung cancer treated with 3D conformal radiotherapy: a meta-analysis.* Clinical Therapeutics, 2015. 37(10): p. 2256-66.
3. Mokhles, S., et al., *Survival and treatment of non-small cell lung cancer stage I-II treated surgically or with stereotactic body radiotherapy: patient and tumor-specific factors affect the prognosis.* Annals of Surgical Oncology, 2015. 22(1): p. 316-23.
4. Louie, A.V., et al., *Predicting Overall Survival After Stereotactic Ablative Radiation Therapy in Early-Stage Lung Cancer: Development and External Validation of the Amsterdam Prognostic Model.* International Journal of Radiation Oncology, Biology, Physics, 2015. 93(1): p. 82-90.
5. Koshy, M., et al., *Increasing radiation therapy dose is associated with improved survival in patients undergoing stereotactic body radiation therapy for stage I non-small-cell lung cancer.* International Journal of Radiation Oncology, Biology, Physics, 2015. 91(2): p. 344-50.
6. Koshy, M., et al., *Stereotactic body radiotherapy and treatment at a high volume facility is associated with improved survival in patients with inoperable stage I non-small cell lung cancer.* Radiotherapy & Oncology, 2015. 114(2): p. 148-54.
7. Jin, J.Y., et al., *Use a survival model to correlate single-nucleotide polymorphisms of DNA repair genes with radiation dose-response in patients with non-small cell lung cancer.* Radiotherapy & Oncology, 2015. 117(1): p. 77-82.
8. Heal, C., et al., *Definitive Treatment of Early-Stage Non-Small Cell Lung Cancer with Stereotactic Ablative Body Radiotherapy in a Community Cancer Center Setting.* Frontiers in Oncology, 2015. 5: p. 146.
9. Russell, K., et al., *Prognostic factors in the radical nonsurgical treatment of stage IIIB non-small-cell lung cancer.* Clinical Lung Cancer, 2014. 15(3): p. 237-43.
10. Ostheimer, C., et al., *A pilot study on potential plasma hypoxia markers in the radiotherapy of non-small cell lung cancer. Osteopontin, carbonic anhydrase IX and vascular endothelial growth factor.* Strahlentherapie und Onkologie, 2014. 190(3): p. 276-82.
11. Koshy, M., et al., *The effect of radiotherapy dose on survival in stage III non-small-cell lung cancer patients undergoing definitive chemoradiotherapy.* Clinical Lung Cancer, 2014. 15(5): p. 365-71.
12. Han, C.B., et al., *Pulmonary artery invasion, high-dose radiation, and overall survival in patients with non-small cell lung cancer.* International Journal of Radiation Oncology, Biology, Physics, 2014. 89(2): p. 313-21.
13. Xue, S.L., et al., *Association between single nucleotide polymorphisms of the transforming growth factor-beta1 gene and overall survival in unresectable locally advanced non-small-cell lung cancer patients treated with radio(chemo)therapy in a Chinese population.* Medical Oncology, 2013. 30(2): p. 512.
14. Mac Manus, M.P., et al., *The use of fused PET/CT images for patient selection and radical radiotherapy target volume definition in patients with non-small cell lung cancer: results of a prospective study with mature survival data.* Radiotherapy & Oncology, 2013. 106(3): p. 292-8.
15. Guckenberger, M., et al., *Lack of a dose-effect relationship for pulmonary function changes after stereotactic body radiation therapy for early-stage non-small cell lung cancer.* International Journal of Radiation Oncology, Biology, Physics, 2013. 85(4): p. 1074-81.
16. Carvalho, S., et al., *Prognostic value of metabolic metrics extracted from baseline positron emission tomography images in non-small cell lung cancer.* Acta Oncologica, 2013. 52(7): p. 1398-404.
17. Ball, D.L., et al., *The complex relationship between lung tumor volume and survival in patients with non-small cell lung cancer treated by definitive radiotherapy: a prospective, observational prognostic factor study of the Trans-Tasman Radiation Oncology Group (TROG 99.05).* Radiotherapy & Oncology, 2013. 106(3): p. 305-11.
18. Ball, D., et al., *Effect of tumor size on prognosis in patients treated with radical radiotherapy or chemoradiotherapy for non-small cell lung cancer. An analysis of the staging project database of the International Association for the Study of Lung Cancer.* Journal of Thoracic Oncology: Official Publication of the International Association for the Study of Lung Cancer, 2013. 8(3): p. 315-21.
19. Aridgides, P.D., et al., *Radiotherapy for stage III non-small-cell lung carcinoma in the elderly (age > 70 years).* Clinical Lung Cancer, 2013. 14(6): p. 674-9.
20. Nawara, C., et al., *The impact of PET and PET/CT on treatment planning and prognosis of patients with NSCLC treated with radiation therapy.* The Quarterly Journal of Nuclear Medicine & Molecular Imaging, 2012. 56(2): p. 191-201.
21. Hamamoto, Y., et al., *Factors affecting survival time after recurrence of non-small-cell lung cancer treated with concurrent chemoradiotherapy.* Japanese Journal of Radiology, 2012. 30(3): p. 249-54.
22. Hallqvist, A., et al., *Mutated KRAS Is an Independent Negative Prognostic Factor for Survival in NSCLC Stage III Disease Treated with High-Dose Radiotherapy.* Lung Cancer International Print, 2012. 2012: p. 587424.
23. Dehing-Oberije, C., et al., *Development and validation of a prognostic model using blood biomarker information for prediction of survival of non-small-cell lung cancer patients treated with combined chemotherapy and radiation or radiotherapy alone (NCT00181519, NCT00573040, and NCT00572325).* International Journal of Radiation Oncology, Biology, Physics, 2011. 81(2): p. 360-8.
24. Siddiqui, F., et al., *The influence of gender, race, and marital status on survival in lung cancer patients: analysis of Radiation Therapy Oncology Group trials.* Journal of Thoracic Oncology: Official Publication of the International Association for the Study of Lung Cancer, 2010. 5(5): p. 631-9.
25. McAleer, M.F., et al., *Does response to induction chemotherapy predict survival for locally advanced non-small-cell lung cancer? Secondary analysis of RTOG 8804/8808.* International Journal of Radiation Oncology, Biology, Physics, 2010. 76(3): p. 802-8.
26. Jayasurya, K., et al., *Comparison of Bayesian network and support vector machine models for two-year survival prediction in lung cancer patients treated with radiotherapy.* Medical Physics, 2010. 37(4): p. 1401-7.
27. Guan, X., et al., *Genotypes and haplotypes of the VEGF gene and survival in locally advanced non-small cell lung cancer patients treated with chemoradiotherapy.* BMC Cancer, 2010. 10: p. 431.
28. Wang, L., et al., *The effect of radiation dose and chemotherapy on overall survival in 237 patients with Stage III non-small-cell lung cancer.* International Journal of Radiation Oncology, Biology, Physics, 2009. 73(5): p. 1383-90.
29. Pemberton, L.S., et al., *Accelerated radical radiotherapy for non-small cell lung cancer using two common regimens: a single-centre retrospective study of outcome.* Clinical Oncology (Royal College of Radiologists), 2009. 21(3): p. 161-7.
30. Kopek, N., et al., *Co-morbidity index predicts for mortality after stereotactic body radiotherapy for medically inoperable early-stage non-small cell lung cancer.* Radiotherapy & Oncology, 2009. 93(3): p. 402-7.
31. Harada, H., et al., *Comparison of chemotherapy regimens for concurrent chemoradiotherapy in unresectable stage III non-small cell lung cancer.* International Journal of Clinical Oncology, 2009. 14(6): p. 507-12.
32. Dehing-Oberije, C., et al., *Development and external validation of prognostic model for 2-year survival of non-small-cell lung cancer patients treated with chemoradiotherapy.* International Journal of Radiation Oncology, Biology, Physics, 2009. 74(2): p. 355-62.
33. Belliere, A., et al., *Feasibility of high-dose three-dimensional radiation therapy in the treatment of localised non-small-cell lung cancer.* Cancer Radiotherapie, 2009. 13(4): p. 298-304.
34. Dehing-Oberije, C., et al., *Tumor volume combined with number of positive lymph node stations is a more important prognostic factor than TNM stage for survival of non-small-cell lung cancer patients treated with (chemo)radiotherapy.* International Journal of Radiation Oncology, Biology, Physics, 2008. 70(4): p. 1039-44.
35. Vokes, E.E., et al., *Induction chemotherapy followed by chemoradiotherapy compared with chemoradiotherapy alone for regionally advanced unresectable stage III Non-small-cell lung cancer: Cancer and Leukemia Group B.* Journal of Clinical Oncology, 2007. 25(13): p. 1698-704.
36. de Cos Escuin, J.S., et al., *[Stage IIIA and IIIB non-small cell lung cancer: results of chemotherapy combined with radiation therapy and analysis of prognostic factors].* Archivos de Bronconeumologia, 2007. 43(7): p. 358-65.
37. Firat, S., et al., *Age is independent of comorbidity influencing patient selection for combined modality therapy for treatment of stage III nonsmall cell lung cancer (NSCLC).* American Journal of Clinical Oncology, 2006. 29(3): p. 252-7.
38. Basaki, K., et al., *Prognostic factors for survival in stage III non-small-cell lung cancer treated with definitive radiation therapy: impact of tumor volume.* International Journal of Radiation Oncology, Biology, Physics, 2006. 64(2): p. 449-54.
39. Pradier, O., et al., *Concurrent low-dose cisplatin and thoracic radiotherapy in patients with inoperable stage III non-small cell lung cancer: a phase II trial with special reference to the hemoglobin level as prognostic parameter.* Journal of Cancer Research & Clinical Oncology, 2005. 131(4): p. 261-9.
40. Bollmann, A., et al., *Survival of patients in clinical stages I-IIIb of non-small-cell lung cancer treated with radiation therapy alone. Results of a population-based study in Southern Saxony-Anhalt.* Strahlentherapie und Onkologie, 2004. 180(8): p. 488-96.
41. Berghmans, T., et al., *Survival is better predicted with a new classification of stage III unresectable non-small cell lung carcinoma treated by chemotherapy and radiotherapy.* Lung Cancer, 2004. 45(3): p. 339-48.
42. Ohe, Y., et al., *Long-term follow-up of patients with unresectable locally advanced non-small cell lung cancer treated with chemoradiotherapy: a retrospective analysis of the data from the Japan Clinical Oncology Group trials (JCOG0003A).* Cancer Science, 2003. 94(8): p. 729-34.
43. Milker-Zabel, S., et al., *Calcification in coronary arteries as quantified by CT scans correlated with tobacco consumption in patients with inoperable non-small cell lung cancer treated with three-dimensional radiotherapy.* British Journal of Radiology, 2003. 76(912): p. 891-6.
44. Mac Manus, M.P., et al., *Positron emission tomography is superior to computed tomography scanning for response-assessment after radical radiotherapy or chemoradiotherapy in patients with non-small-cell lung cancer.* Journal of Clinical Oncology, 2003. 21(7): p. 1285-92.
45. MacRae, R., et al., *Declining hemoglobin during chemoradiotherapy for locally advanced non-small cell lung cancer is significant.* Radiotherapy & Oncology, 2002. 64(1): p. 37-40.
46. Firat, S., R.W. Byhardt, and E. Gore, *Comorbidity and Karnofksy performance score are independent prognostic factors in stage III non-small-cell lung cancer: an institutional analysis of patients treated on four RTOG studies. Radiation Therapy Oncology Group.* International Journal of Radiation Oncology, Biology, Physics, 2002. 54(2): p. 357-64.
47. Ball, D., et al., *Failure of T stage to predict survival in patients with non-small-cell lung cancer treated by radiotherapy with or without concomitant chemotherapy.* International Journal of Radiation Oncology, Biology, Physics, 2002. 54(4): p. 1007-13.
48. Werner-Wasik, M., et al., *Recursive partitioning analysis of 1999 Radiation Therapy Oncology Group (RTOG) patients with locally-advanced non-small-cell lung cancer (LA-NSCLC): identification of five groups with different survival.* International Journal of Radiation Oncology, Biology, Physics, 2000. 48(5): p. 1475-82.
49. Langendijk, H., et al., *The prognostic impact of quality of life assessed with the EORTC QLQ-C30 in inoperable non-small cell lung carcinoma treated with radiotherapy.* Radiotherapy & Oncology, 2000. 55(1): p. 19-25.
50. Erkurt, E., C. Tunali, and M. Erkisi, *Primary therapeutic decision-making in inoperable non-small cell lung cancer.* International Journal of Radiation Oncology, Biology, Physics, 2000. 46(2): p. 439-44.
51. Jeremic, B., et al., *Hyperfractionated radiotherapy for clinical stage II non-small cell lung cancer.* Radiotherapy & Oncology, 1999. 51(2): p. 141-5.
52. Mirimanoff, R.O., et al., *Alternating radiotherapy and chemotherapy for inoperable Stage III non-small-cell lung cancer: long-term results of two Phase II GOTHA trials. Groupe d'Oncologie Thoracique Alpine.* International Journal of Radiation Oncology, Biology, Physics, 1998. 42(3): p. 487-94.
53. Komaki, R., et al., *Failure patterns by prognostic group determined by recursive partitioning analysis (RPA) of 1547 patients on four radiation therapy oncology group (RTOG) studies in inoperable nonsmall-cell lung cancer (NSCLC).* International Journal of Radiation Oncology, Biology, Physics, 1998. 42(2): p. 263-7.
54. Wigren, T., H. Oksanen, and P. Kellokumpu-Lehtinen, *A practical prognostic index for inoperable non-small-cell lung cancer.* Journal of Cancer Research & Clinical Oncology, 1997. 123(5): p. 259-66.
55. Wigren, T., *Confirmation of a prognostic index for patients with inoperable non-small cell lung cancer.* Radiotherapy & Oncology, 1997. 44(1): p. 9-15.
56. Scott, C., et al., *Recursive partitioning analysis of 1592 patients on four Radiation Therapy Oncology Group studies in inoperable non-small cell lung cancer.* Lung Cancer, 1997. 17 Suppl 1: p. S59-74.
57. Reboul, F., et al., *Concurrent cisplatin, etoposide, and radiotherapy for unresectable stage III nonsmall cell lung cancer: a phase II study.* International Journal of Radiation Oncology, Biology, Physics, 1996. 35(2): p. 343-50.
58. Wurschmidt, F., et al., *Inoperable non-small cell lung cancer: a retrospective analysis of 427 patients treated with high-dose radiotherapy.* International Journal of Radiation Oncology, Biology, Physics, 1994. 28(3): p. 583-8.
59. Hayakawa, K., et al., *Effect of krestin (PSK) as adjuvant treatment on the prognosis after radical radiotherapy in patients with non-small cell lung cancer.* Anticancer Research, 1993. 13(5C): p. 1815-20.
60. Cox, J.D., et al., *Interruptions of high-dose radiation therapy decrease long-term survival of favorable patients with unresectable non-small cell carcinoma of the lung: analysis of 1244 cases from 3 Radiation Therapy Oncology Group (RTOG) trials.* International Journal of Radiation Oncology, Biology, Physics, 1993. 27(3): p. 493-8.
61. Thorogood, J., et al., *The use of discriminant analysis to guide palliative treatment for lung cancer patients.* Clinical Oncology (Royal College of Radiologists), 1992. 4(1): p. 22-6.
62. Robinow, J.S., et al., *Results of combination chemotherapy and thoracic radiation therapy for unresectable non-small cell carcinoma of the lung.* International Journal of Radiation Oncology, Biology, Physics, 1989. 17(6): p. 1203-10.
63. Kaasa, S., A. Mastekaasa, and E. Lund, *Prognostic factors for patients with inoperable non-small cell lung cancer, limited disease. The importance of patients' subjective experience of disease and psychosocial well-being.* Radiotherapy & Oncology, 1989. 15(3): p. 235-42.
64. Yang, P., et al., *Analysis of the correlation among hypertension, the intake of beta-blockers, and overall survival outcome in patients undergoing chemoradiotherapy with inoperable stage III non-small cell lung cancer.* American Journal of Cancer Research, 2017. 7: p. 946-954.
65. Yu, Y., et al., *Expression of raf kinase inhibitor protein and radiotherapy prognosis of non-small-cell lung cancer.* International Journal of Clinical and Experimental Medicine, 2017. 10: p. 3502-3509.
66. Ahmed, H.Z., et al., *Guideline-concordant Care Improves Overall Survival for Locally Advanced Non-Small-cell Lung Carcinoma Patients: A National Cancer Database Analysis.* Clin Lung Cancer, 2017.
67. Eschmann, S.M., et al., *Is standardised 18F-FDG uptake value an outcome predictor in patients with stage III non-small cell lung cancer?* European Journal of Nuclear Medicine and Molecular Imaging, 2006. 33: p. 263-269.
68. Choy, H., et al., *Phase II multicenter study of induction chemotherapy followed by concurrent efaproxiral (RSR13) and thoracic radiotherapy for patients with locally advanced non-small-cell lung cancer.* Journal of Clinical Oncology, 2005. 23: p. 5918-5928.
69. Harris, J.P., et al., *A population-based comparative effectiveness study of chemoradiation regimens and sequences in stage III non-small cell lung cancer.* Lung Cancer, 2017. 108: p. 173-182.
70. Urvay, S.E., et al., *Prognostic Factors in Stage III Non-Small-Cell Lung Cancer Patients.* Asian Pac J Cancer Prev, 2016. 17: p. 4693-4697.
71. Imai, H., et al., *Prognostic significance of diabetes mellitus in locally advanced non-small cell lung cancer.* BMC Cancer, 2015. 15 (1) (no pagination).
72. Saito, Y., et al., *Prognostic value of pretreatment serum carcinoembryonic antigen and squamous cell carcinoma antigen levels for patients with stage I-III non- small cell lung cancer treated with radiation therapy alone.* International Journal of Clinical Oncology, 1998. 3: p. 27-30.
73. Ghosal, N., et al., *Radical hypofractionated radiotherapy for the treatment of non-small-cell lung cancer using 52.5-55 Gy in 20 fractions: The North Wales Cancer Centre experience.* Journal of Radiotherapy in Practice, 2015. 14: p. 268-273.
74. Dudani, S., et al., *Radical Treatment of Stage II Non-small-cell Lung Cancer With Nonsurgical Approaches: A Multi-institution Report of Outcomes.* Clin Lung Cancer, 2017.
75. Sanchez De Cos Escuin, J., et al., *Stage IIIA and IIIB non-small cell lung cancer: Results of chemotherapy combined with radiation therapy and analysis of prognostic factors Carcinoma de pulmon no microcitico. Estadios IIIA y B. Resultados del tratamiento combinado (quimioterapia y radioterapia) y analisis de factores pronosticos.* Archivos de Bronconeumologia, 2007. 43: p. 358-365.
76. Stinchcombe, T.E., et al., *Treatment outcomes of different prognostic groups of patients on Cancer and Leukemia Group B trial 39801: Induction chemotherapy followed by chemoradiotherapy compared with chemoradiotherapy alone for unresectable stage III non-small cell lung cancer.* Journal of Thoracic Oncology, 2009. 4: p. 1117-1125.
77. Han, Y., et al., *X-radiation inhibits histone deacetylase 1 and 2, upregulates Axin expression and induces apoptosis in non-small cell lung cancer.* Radiation Oncology, 2012. 7 (1) (no pagination).

- **Reason for record exclusion:** reports excluded because none of the risk factors evaluated are factors that were measured at or before the start of RRT; N = 3 [265-267]

1. Kim, D.W., et al., *Response to combined modality therapy correlates with survival in locally advanced non-small-cell lung cancer.* International Journal of Radiation Oncology, Biology, Physics, 2005. 63(4): p. 1029-36.
2. Arpin, D., et al., *Early variations of circulating interleukin-6 and interleukin-10 levels during thoracic radiotherapy are predictive for radiation pneumonitis.* Journal of Clinical Oncology, 2005. 23: p. 8748-8756.
3. Cremonesi, M., et al., *Role of interim 18F-FDG-PET/CT for the early prediction of clinical outcomes of Non-Small Cell Lung Cancer (NSCLC) during radiotherapy or chemo-radiotherapy. A systematic review.* Eur J Nucl Med Mol Imaging, 2017.

- **Reason for record exclusion:** reports excluded because evaluated RRT dose (but not the modality of RRT) as the only risk factor for response to treatment; N = 16 [268-283]

1. Santiago, A., et al., *Challenges in radiobiological modeling: can we decide between LQ and LQ-L models based on reviewed clinical NSCLC treatment outcome data?* Radiation Oncology, 2016. 11(1): p. 67.
2. Rodrigues, G., et al., *Is intermediate radiation dose escalation with concurrent chemotherapy for stage III non-small-cell lung cancer beneficial? A multi-institutional propensity score matched analysis.[Erratum appears in Int J Radiat Oncol Biol Phys. 2015 Apr 1;91(5):1115; PMID: 25832709].* International Journal of Radiation Oncology, Biology, Physics, 2015. 91(1): p. 133-9.
3. Chetty, I.J., et al., *Correlation of dose computed using different algorithms with local control following stereotactic ablative radiotherapy (SABR)-based treatment of non-small-cell lung cancer.* Radiotherapy & Oncology, 2013. 109(3): p. 498-504.
4. Wang, D., et al., *Functional dosimetric metrics for predicting radiation-induced lung injury in non-small cell lung cancer patients treated with chemoradiotherapy.* Radiation Oncology, 2012. 7: p. 69.
5. Ohri, N., et al., *Modeling local control after hypofractionated stereotactic body radiation therapy for stage I non-small cell lung cancer: a report from the elekta collaborative lung research group.* International Journal of Radiation Oncology, Biology, Physics, 2012. 84(3): p. e379-84.
6. Kwint, M., et al., *Acute esophagus toxicity in lung cancer patients after intensity modulated radiation therapy and concurrent chemotherapy.* International Journal of Radiation Oncology, Biology, Physics, 2012. 84(2): p. e223-8.
7. Huang, E.X., et al., *Modeling the risk of radiation-induced acute esophagitis for combined Washington University and RTOG trial 93-11 lung cancer patients.* International Journal of Radiation Oncology, Biology, Physics, 2012. 82(5): p. 1674-9.
8. Tucker, S.L., et al., *Impact of toxicity grade and scoring system on the relationship between mean lung dose and risk of radiation pneumonitis in a large cohort of patients with non-small cell lung cancer.* International Journal of Radiation Oncology, Biology, Physics, 2010. 77(3): p. 691-8.
9. Ramella, S., et al., *Adding ipsilateral V20 and V30 to conventional dosimetric constraints predicts radiation pneumonitis in stage IIIA-B NSCLC treated with combined-modality therapy.* International Journal of Radiation Oncology, Biology, Physics, 2010. 76(1): p. 110-5.
10. Barriger, R.B., et al., *Dose-volume analysis of radiation pneumonitis in non-small-cell lung cancer patients treated with concurrent cisplatinum and etoposide with or without consolidation docetaxel.* International Journal of Radiation Oncology, Biology, Physics, 2010. 78(5): p. 1381-6.
11. Koto, M., et al., *Dosimetric factors used for thoracic X-ray radiotherapy are not predictive of the occurrence of radiation pneumonitis after carbon-ion radiotherapy.* Tohoku Journal of Experimental Medicine, 2007. 213(2): p. 149-56.
12. Rosenzweig, K.E., et al., *Results of a phase I dose-escalation study using three-dimensional conformal radiotherapy in the treatment of inoperable nonsmall cell lung carcinoma.* Cancer, 2005. 103(10): p. 2118-27.
13. Mehta, M., et al., *A new approach to dose escalation in non-small-cell lung cancer.* International Journal of Radiation Oncology, Biology, Physics, 2001. 49(1): p. 23-33.
14. Singer, J.M., P. Price, and R.G. Dale, *Radiobiological prediction of normal tissue toxicities and tumour response in the radiotherapy of advanced non-small-cell lung cancer.* British Journal of Cancer, 1998. 78(12): p. 1629-33.
15. Roach, I.M., et al., *Radiation pneumonitis following combined modality therapy for lung cancer: Analysis of prognostic factors.* Journal of Clinical Oncology, 1995. 13: p. 2606-2612.
16. Davis, J.N., et al., *Stereotactic body radiotherapy for early-stage non-small cell lung cancer: clinical outcomes from a National Patient Registry.* J Radiat Oncol, 2015. 4: p. 55-63.

- **Reason for record exclusion:** model development methods paper using radiotherapy for NSCLC as an example; results not intended as formal clinical findings; N = 2 [284, 285]

1. Lindblom, E., A. Dasu, and I. Toma-Dasu, *Optimal fractionation in radiotherapy for non-small cell lung cancer--a modelling approach.* Acta Oncologica, 2015. 54(9): p. 1592-8.
2. Naqa, I.E., et al., *Datamining approaches for modeling tumor control probability.* Acta Oncologica, 2010. 49(8): p. 1363-73.

- **Reason for record exclusion:** unclear record; N = 30 [286-315]

1. Tucker, S.L., et al., *Predicting pneumonitis risk: a dosimetric alternative to mean lung dose.* International Journal of Radiation Oncology, Biology, Physics, 2013. 85(2): p. 522-7.
2. Strigari, L., et al., *A modified hypoxia-based TCP model to investigate the clinical outcome of stereotactic hypofractionated regimes for early stage non-small-cell lung cancer (NSCLC).* Medical Physics, 2012. 39(7): p. 4502-14.
3. Kyas, I., et al., *Prediction of radiation-induced changes in the lung after stereotactic body radiation therapy of non-small-cell lung cancer.* International Journal of Radiation Oncology, Biology, Physics, 2007. 67(3): p. 768-74.
4. Clement, D., L. Miron, and M. Marinca, *[Age-related prognostic factors and treatment results for advanced non-small cell lung cancer (NSCLC)].* Revista Medico-Chirurgicala a Societatii de Medici Si Naturalisti Din Iasi, 2007. 111(4): p. 856-63.
5. Bradley, J.D., et al., *A nomogram to predict radiation pneumonitis, derived from a combined analysis of RTOG 9311 and institutional data.* International Journal of Radiation Oncology, Biology, Physics, 2007. 69(4): p. 985-92.
6. Hope, A.J., et al., *Modeling radiation pneumonitis risk with clinical, dosimetric, and spatial parameters.* International Journal of Radiation Oncology, Biology, Physics, 2006. 65(1): p. 112-24.
7. Hayashi, M., et al., *Automatic search for optimal conditions in clinical studies.* Anticancer Research, 2001. 21(2B): p. 1371-4.
8. Coen, V., et al., *Prognostic factors in locoregional non-small cell lung cancer treated with radiotherapy.* American Journal of Clinical Oncology, 1995. 18(2): p. 111-7.
9. Leung, W.T., et al., *Combined chemotherapy and radiotherapy versus best supportive care in the treatment of inoperable non-small-cell lung cancer.* Oncology, 1992. 49(5): p. 321-6.
10. Pluta, E., *Analysis of prognostic factors in patients with non-small cell lung cancer treated conventional radical teleradiotherapy Ocena czynnikow prognostycznych u chorych na niedrobnokomo rkowego raka pluca po radykalnej radioterapii konwencjonalnej.* Nowotwory, 2007. 57: p. 533-541.
11. Yu, E., et al., *Definitive radiation therapy management for medically non-resectable clinically localised non-small cell lung cancer: Results & prognostic factors Radykalna radioterapia nieoperacyjnego ze wzgledow medycznych, klinicznie miejscowo ograniczonego, niedrobnokomorkowego raka pluc: Wyniki i czynniki prognostyczne.* Nowotwory, 2007. 57: p. 646-653.
12. Chmielewska, E., et al., *Efficacy and tolerance of palliative accelerated radiation therapy combined with chemotherapy in inoperable non-small cell lung cancer patients - Retrospective analysis Wartosc przyspieszonej radioterapii stosowanej wraz z chemioterapia u pacjentow z niedrobnokomorkowym, nieoperacyjnym rakiem pluca - Analiza retrospektywna.* Nowotwory, 2008. 58: p. 133-137.
13. Reinfuss, M., et al., *Evaluation of efficacy of combined chemoradiotherapy in locoregional advanced, inoperable Non-Small Cell Lung Cancer (clinical randomized trial).* Nowotwory, 2005. 55: p. 200-206.
14. Bernchou, U., et al., *Extent and computed tomography appearance of early radiation induced lung injury for non-small cell lung cancer.* Radiother Oncol, 2017. 123: p. 93-98.
15. Xia, H., et al., *EZH2 silencing with RNAi enhances irradiation-induced inhibition of human lung cancer growth in vitro and in vivo.* Oncol Lett, 2012. 4: p. 135-140.
16. Wurschmidt, F., et al., *High-dose radiotherapy of inoperable non-small cell lung cancer. A retrospective analysis of 427 patients Strahlentherapie Des Inoperable Nichtkleinzelligen Bronchialkarzinoms. Eine Retrospektive Analyse Von 427 Fallen.* Strahlentherapie und Onkologie, 1994. 170: p. 302-304.
17. Aich, R.K., et al., *Hypofractionated radiotherapy (MRC trial) - A preferred schedule for locally advanced non-small cell lung cancers.* Indian Journal of Radiology and Imaging, 1998. 8: p. 177-181.
18. Huang, E.X., et al., *Independent test of a model to predict severe acute esophagitis.* Advances in Radiation Oncology, 2017. 2: p. 37-43.
19. Kim, J.H., et al., *Outcome of active anti-cancer treatment in elderly patients with advanced non-small cell lung cancer: A single center experience.* Thoracic Cancer, 2014. 5: p. 133-138.
20. *(P101) Does Maximum SUV From F-18 PET Scan Predict Outcomes for Early-Stage Non-Small-Cell Lung Cancer Treated With Stereotactic Body Radiotherapy (SBRT)?* Oncology (Williston Park), 2015. 29.
21. Yang, J., et al., *Polymorphisms in BMP2/BMP4, with estimates of mean lung dose, predict radiation pneumonitis among patients receiving definitive radiotherapy for non-small cell lung cancer.* Oncotarget, 2017. 8: p. 43080-43090.
22. Giuliani, M.E., et al., *Predictors and Patterns of Regional Recurrence Following Lung SBRT: A Report From the Elekta Lung Research Group.* Clinical Lung Cancer, 2017. 18: p. 162-168.
23. Yalman, D., et al., *Radical radiotherapy in advanced-stage non-small cell lung cancer: Evaluation of 332 cases.* Journal of B.U.ON., 1999. 4: p. 383-387.
24. Yamazaki, H., et al., *Radiographic changes following radiotherapy in the patients with lung cancer. Is the irradiated area of the mediastinum in the simulation film a significant factor?* Strahlentherapie und Onkologie, 1995. 171: p. 272-7.
25. Matsumoto, Y., et al., *Radiotherapy alone versus chemoradiotherapy in nonresectable non-small cell lung cancer; analysis of a multi-institutional study.* Japanese Journal of Clinical Radiology, 1997. 42: p. 1705-1710.
26. Linam, J.M., et al., *The relationship between pulmonary function metrics and radiation-induced lung injury.* Journal of Solid Tumors, 2013. 3: p. 6-13.
27. Inagaki, M., et al., *S-1-containing chemotherapy for patients with non-small-cell lung cancer: A population-based observational study by the ibaraki thoracic integrative (POSITIVE) research group.* Molecular and Clinical Oncology, 2016. 4: p. 1025-1030.
28. Cassidy, R.J., et al., *Stereotactic Body Radiotherapy for Early-stage Non-small-cell Lung Cancer in Patients 80 Years and Older: A Multi-center Analysis.* Clin Lung Cancer, 2017.
29. Krafft, S., et al., *SU-F-BRCD-05: Mean Regional Dose to the Esophagus Predicts Acute Toxicity Rate for Lung Cancer Patients.* Med Phys, 2012. 39: p. 3856.
30. Rocha Lima, C.M.S., et al., *Therapy choices among older patients with lung carcinoma: An evaluation of two trials of the cancer and leukemia group B.* Cancer, 2002. 94: p. 181-187.

- **Reason for record exclusion:** ongoing trials*;* N = 2

1. Bogart, J.A. and R. Govindan, A randomized phase II study of radiation therapy, pemetrexed, and carboplatin with or without cetuximab in stage III non-small-cell lung cancer. Clinical Lung Cancer, 2006. 7: p. 285-287.
2. Specht, H.M., et al., Heat shock protein 70 (Hsp70) peptide activated Natural Killer (NK) cells for the treatment of patients with non-small cell lung cancer (NSCLC) after radiochemotherapy (RCTx) - From preclinical studies to a clinical phase II trial. Frontiers in Immunology, 2015. 6 (MAR) (no pagination).
