# Supplementary material for: Predicting Outcomes From Radical Radiotherapy for Non-small Cell Lung Cancer: A Systematic Review of the Existing Literature
Source: Front Oncol. 2018 Oct 10;8:433. doi: 10.3389/fonc.2018.00433 (PMC6191477; doi:10.3389/fonc.2018.00433)
Supplement: Supplementary file 3 [file Data_Sheet_3.DOCX]

Appendix C

**Figure 1.** N of included reports by year of publication

**Table 1.** Mapping of radical radiotherapy treatment (total N of included study reports=259)

| **Item** | **Variables** | **N** | **%** |
| --- | --- | --- | --- |
| **Radiation type** |  |  |  |
|  | Protons | 13 | 5 |
|  | Electrons | 2 | 1 |
|  | Photons | 83 | 32 |
|  | Unclear or NR | 161 | 62 |
| **Mode of radiotherapy** |  |  |  |
|  | *2D conventional* | 8 | 3 |
|  | *3D conformal* | 65 | 25 |
|  | *IMRT* | 8 | 3 |
|  | *VMAT* | 2 | 1 |
|  | *Stereotactic ablative* | 47 | 18 |
|  | Multiple RT techniques  (commonly mix of 3D conformal and IMRT) | 42 | 16 |
|  | *NR* | 87 | 34 |
| **Fractionation** |  |  |  |
|  | *Conventional* | 48 | 19 |
|  | *Dose-intensified conventional* | 18 | 7 |
|  | *Hypofractionation* | 66 | 25 |
|  | *Hyperfractionation* | 19 | 7 |
|  | *Mixed fractionation protocols* | 79 | 31 |
|  | *NR* | 29 | 11 |

IMRT=intensity modulated radiation therapy; N=number of study reports; VMAT= volumetric modulated arc therapy; NR=not reported; RT=radiotherapy

**Table 2.** Mapping of chemotherapy treatment (total N of included study reports=259)

| **Item** | **Variables** | **N** | **%** |
| --- | --- | --- | --- |
| **Chemotherapy** |  |  |  |
|  | *No chemotherapy was received by any study patient* | 80 | 31 |
|  | *A proportion of study patients received one or more types of chemotherapy (sequential, concurrent, adjuvant or combinations of these)* | 88 | 34 |
|  | *All study patients received sequential chemotherapy as the only chemotherapy* | 7 | 3 |
|  | *All study patients received concurrent chemotherapy as the only chemotherapy* | 33 | 13 |
|  | *All study patients received adjuvant chemotherapy as the only chemotherapy* | 1 | <1 |
|  | *All study patients received sequential and concurrent chemotherapy* | 4 | <1 |
|  | *All study patients received concurrent and adjuvant chemotherapy* | 2 | <1 |
|  | *All study patients received sequential and adjuvant chemotherapy* | 1 | <1 |
|  | *All study patients received sequential plus concurrent plus adjuvant chemotherapy* | 0 | 0 |
|  | *All study patients receive chemotherapy, but type of chemotherapy (i.e. sequential, concurrent, adjuvant, or combinations) varies across patients* | 43 | 17 |

N=number of study reports
