# Supplementary material for: Predicting Outcomes From Radical Radiotherapy for Non-small Cell Lung Cancer: A Systematic Review of the Existing Literature
Source: Front Oncol. 2018 Oct 10;8:433. doi: 10.3389/fonc.2018.00433 (PMC6191477; doi:10.3389/fonc.2018.00433)
Supplement: Supplementary file 4 [file Data_Sheet_4.DOCX]

Appendix D

Table of included prognostic model research

| Author, year | N | Population & design | Radiotherapy | Chemotherapy | Predicted outcomes | Final model factors | Model risk of bias | RoB support | Does the model investigate differential treatment response? |
| --- | --- | --- | --- | --- | --- | --- | --- | --- | --- |
| Salama, 2011 [1] | 68 | Inoperable stage III NSCLC  Data from an RCT of two different chemotherapy protocols | 3DCRT  Conventional dose intensified | Induction + concurrent (carboplatin + paclitaxel vs. gemcitabine) | Composite lung toxicity (≥grade 3) | V20 and nodal stage | High | No discrimination statistics reported, small number of events, no final model formula was reported, not externally validated. | No |
| Wijsman, 2015 [2] | 149 | Advanced stage or inoperable NSCLC  (PET-staged)  Non-comparative cohort | IMRT or VMAT conventional fractionation | Concurrent, sequential, or no chemotherapy | Acute oesophageal toxicity | Type of chemotherapy; advance clinical stage, gender and mean esophageal dose | High | Attrition bias, retrospective assessment of toxicity and dose-volume histogram, and lack of external validation | No |
| Fried, 2014 [3] | 91 | Stage III NSCLC patients undergoing definitive chemoradiation  Non-comparative cohort | 3DCRT or IMRT  Conventional fractionation | Concurrent ± adjuvant | LC  FDM | LC: CT and 4DCT based texture features  FDM: gender, GTV and CT and 4DCT based texture features | High | Selection bias, retrospective non-blinded texture analysis, treatment factor not included in the model, no model calibration, no external validation, and no final model formula was reported | No |
| Palma, 2013 [4] | 836 | Patients with NSCLC treated with concurrent chemoradiotherapy  Systematic review which included an individual patient data meta-analysis of non-reported study designs | 3DCRT or IMRT | Concurrent ± sequential/adjuvant | Symptomatic radiation  pneumonitis (≥ grade 2) | Lung V20, chemotherapy regimen, and age | High | Publication bias, lack of critical appraisal of the validity and design of contributing studies, risk of attrition bias because of missing data, non-reporting of final formula and calibration statistics | No |
| Pan, 2016 [5] | 117 | Data from multi-centre RCT including stage III NSCLC patients | Conventional IMRT | Induction + concurrent chemotherapy | Acute oesophagitis (≤ 3 months) | Two final models were optimal:  1) Gender, treating institution, P20, L40  2) Gender, treating institution, P20, oesophagus V40 | High | Model was not externally validated | No |
| Ataman, 2001 [6] | 549 | Competing risk analysis of data from multi-centre RCT including stage I-III NSCLC patients | CHART vs. conventional RT | NR | Local failure &  Distant failure | For both outcomes: age, gender, clinical stage and treatment type.  Advanced clinical stage was associated with a decreased interval to local or distant failure, with a higher risk of failing in distant position | High | Did not report discrimination statistics  Not externally validated | No |
| Lee, 2015 [7] | 54 | Stage III NSCLC patients  Proof of concept evaluation of Bayesian network as a graphical model for modelling joint probability distribution among random variables via a directed acyclic graph – cohort design | 3DCRT | Sequential or concurrent chemotherapy | Radiation pneumonitis | A mix of pre-treatment and mid-treatment factors were included in the model so the study was considered of little clinical relevance | High | Small number of radiation pneumonitis events (N=19), variability in chemotherapy was not factored, no calibration statistics were reported, final model formula was not reported or referenced, and findings have not been externally validated | No |
| Oh, 2011 [8] | 56 (retrospective dataset)  &  18 (prospective dataset) | Locally advanced NSCLC  Proof of concept evaluation of Bayesian network as a graphical model for modelling joint probability distribution among random variables via a directed acyclic graph - cohort | 3DCRT (retrospective)  NR (prospective) | Sequential, concurrent, or no chemotherapy | Local failure | A mix of dosimetric, clinical and mid-treatment biologic markers were evaluated | High | Risk of attrition bias, lack of model formula reporting, non-reporting of calibration statistics, small number of outcomes compared to predictors in the model, and lack of external validation | No |
| Mörth, 2016 [9] | 71 | Patients with locally advanced NSCLC treated with concurrent chemoradiotherapy  Retrospective cohort external validation study of Palma et al.’s risk prediction model | 3DCRT | Concurrent (paclitaxel or platinum-based) + corticosteroids | Symptomatic radiation  pneumonitis (≥ grade 2)  Although no patients deemed high risk as per Palma et al.’s model were found to be in the cohort, 16 patients developed radiation pneumonitis | Palma et al.’s risk prediction model was externally validated: AUC = 0.68 (95% CI, 0.53, 0.82)  Addition of current smoking status increased discrimination to AUC of 0.72 | High | Retrospective and unblinded outcome ascertainment; small number of events, and calibration in the small was not formally assessed | No |
| Li 2017 [10] | 92 | Patients with stage I or II NSCLC planned to undergo stereotactic body radiotherapy  Retrospective cohort | 3DCRT or volumetric arc therapy | No chemotherapy | Recurrence free survival  &  Loco-regional recurrence free survival | Final model included clinical (tumour vessel attachment or ECOG score) and computer derived image feature (short axis × longest diameter) with Harrell’s C-index of 0.61 and 0.66, respectively | High | High risk of selection bias because 42 patients without follow-up data were excluded. Also, models were not externally validated. | No |

**References**

1. Salama, J.K., et al., Pulmonary toxicity in Stage III non-small cell lung cancer patients treated with high-dose (74 Gy) 3-dimensional conformal thoracic radiotherapy and concurrent chemotherapy following induction chemotherapy: a secondary analysis of Cancer and Leukemia Group B (CALGB) trial 30105. International Journal of Radiation Oncology, Biology, Physics, 2011. 81(4): p. e269-74.
2. Wijsman, R., et al., Multivariable normal-tissue complication modeling of acute esophageal toxicity in advanced stage non-small cell lung cancer patients treated with intensity-modulated (chemo-)radiotherapy. Radiotherapy & Oncology, 2015. 117(1): p. 49-54.
3. Fried, D.V., et al., Prognostic value and reproducibility of pretreatment CT texture features in stage III non-small cell lung cancer. International Journal of Radiation Oncology, Biology, Physics, 2014. 90(4): p. 834-42.
4. Palma, D.A., et al., Predicting radiation pneumonitis after chemoradiation therapy for lung cancer: an international individual patient data meta-analysis. International Journal of Radiation Oncology, Biology, Physics, 2013. 85(2): p. 444-50.
5. Pan, Y., et al., Acute esophagitis for patients with local-regional advanced non small cell lung cancer treated with concurrent chemoradiotherapy. Radiotherapy & Oncology, 2016. 118(3): p. 465-70.
6. Ataman, O.U., et al., Failure-specific prognostic factors after continuous hyperfractionated accelerated radiotherapy (CHART) or conventional radiotherapy in locally advanced non-small-cell lung cancer: a competing risks analysis. British Journal of Cancer, 2001. 85(8): p. 1113-8.
7. Lee, S., et al., Bayesian network ensemble as a multivariate strategy to predict radiation pneumonitis risk. Medical Physics, 2015. 42(5): p. 2421-30.
8. Oh, J.H., et al., A Bayesian network approach for modeling local failure in lung cancer. Physics in Medicine & Biology, 2011. 56(6): p. 1635-51.
9. Morth, C., et al., Validation and optimization of a predictive model for radiation pneumonitis in patients with lung cancer. Oncol Lett, 2016. 12: p. 1144-1148.
10. Li, Q., et al., Imaging features from pretreatment CT scans are associated with clinical outcomes in nonsmall-cell lung cancer patients treated with stereotactic body radiotherapy. Med Phys, 2017.
